# Measles virus reprograms CD4^+^ T-cell sphingolipid and fatty acid metabolism to facilitate infection

**DOI:** 10.3389/fcell.2026.1938931

**Published:** 2026-08-19

**Authors:** Maria Fernanda Grijalva Yépez, Yann Loïc Cordes, Agnes Fekete, Fabian Schumacher, Burkhard Kleuser, Elita Avota

**Affiliations:** 1 Institute for Virology and Immunobiology, University of Wuerzburg, Wuerzburg, Germany; 2 Pharmaceutical Biology, Julius-von-Sachs-Institute, Biocenter, University of Wuerzburg, Wuerzburg, Germany; 3 Institute of Pharmacy, Department of Pharmacology, Freie Universität Berlin, Berlin, Germany

**Keywords:** CD4^+^CD150^+^ T cell, ceramide (CER), dihydroceramide (dhCer), hexosylceramide (HexCer), measles virus (MV), signaling lymphocytic activation molecule family member 1 (SLAMF1/CD150), sphingolipids, triacylglycerol (TG)

## Abstract

**Introduction:**

CD150-positive (CD150^+^) lymphocytes are the primary targets of measles virus (MV) infection during the acute phase. The depletion of infected CD150^+^ memory cells leads to a profound suppression of immune memory, resulting in increased susceptibility to secondary infections and severe complications in infected individuals. While lipids are known to be critical for viral life cycles, the specific lipid metabolic conditions facilitating MV replication in immune cells remain poorly understood.

**Methods and Results:**

In this study, a comprehensive lipidomic analysis of MV-infected primary CD4^+^ T cells revealed significant alterations in three major lipid classes: sphingolipids, triacylglycerols, and glycerophospholipids. Specifically, MV infection increased the abundance of ceramides, dihydroceramides and triacylglycerols, while significantly reducing the levels of glucosylceramides and glycerophospholipids. Pharmacological intervention in the pathways of *de novo* sphingolipid synthesis, triacylglycerol synthesis and lipolysis significantly impaired measles virus replication in activated CD4^+^ CD150^+^ T cells.

**Discussion:**

Mechanistically, we demonstrate that MV glycoprotein-mediated membrane fusion, essential for viral entry and cell-to-cell spread, requires glucosylceramide synthase (GCS) activity and is further promoted by plasma membrane triacylglycerol content. Collectively, these findings highlight the essential role of MV-modulated triacylglycerol and sphingolipid metabolism in viral entry, dissemination, and intracellular replication within CD4^+^ T cells.

## 1 Introduction

Measles virus (MV) is highly contagious, enveloped, negative-sense RNA virus belonging to the family *Paramyxoviridae*. Although it spreads through respiratory droplets, MV does not directly infect respiratory epithelial cells upon initial entry. Instead, the primary targets of MV are CD150-positive immature pulmonary dendritic cells (DCs) and alveolar macrophages within the airways. These cells cross the epithelium and transport the virus to draining lymph nodes, where it infects other CD150-expressing cells (Allen et al., 2018; Ferreira et al., 2010; Lemon et al., 2011). CD150/SLAMF1 (Signaling Lymphocytic Activation Molecule Family Member 1), a member of the immunoglobulin superfamily of cell-surface proteins expressed within the hematopoietic compartment, serves as the entry receptor for wild-type MV (Tatsuo et al., 2000; de Swart et al., 2007; Erlenhoefer et al., 2001). It is primarily found on DCs, macrophages, and activated T and B cells. Unlike naïve T cells, which upregulate CD150 only upon activation, memory T cells express it constitutively. Due to the higher baseline expression, memory T cells represent the preferred target for infection within the T-cell compartment (Laksono et al., 2018; Lin et al., 2020). Because of this viral tropism for CD150^+^ memory immune cells, adaptive immunity against previously encountered pathogens is transiently erased upon systemic infection, resulting in profound immunosuppression (de Vries et al., 2012; de Vries and de Swart, 2014; Griffin, 2021). This MV-induced immune amnesia is well-documented in epidemiological studies and correlates with an elevated risk of morbidity and mortality for years following the initial infection (Mina et al., 2019; Petrova et al., 2019; Behrens et al., 2020; Gadroen et al., 2018). While safe and protective vaccination remains the most effective way to prevent complications and immunosuppression, global measles incidence significantly increased in 2024 due to a decline in vaccination rates during the COVID-19 pandemic. The trend persisted into 2025, reaching the highest number of cases worldwide in over 25 years (Do and Mulholland, 2025; Basch et al., 2025; Au et al., 2026). Consequently, the development of novel antiviral interventions remains crucial in MV research.

The manipulation of host lipid metabolism has been documented for a plethora of viruses, enabling them to optimize cellular resources for the production of viral progeny. This establishes a complex interplay between the virus and host lipids, as lipid mediators not only support viral morphogenesis but also regulate antiviral responses and inflammation (Yang et al., 2026). While general trends exist regarding the lipid classes affected during viral infections (Hehner et al., 2024), lipids can be differentially utilized depending on the specific virus. Ongoing comparative studies into the mechanisms of infection-mediated lipid modulation, alongside investigations into their pro- or anti-viral characteristics, continue to reveal virus-specific adaptations tailored to distinct viral life cycles. Among these, sphingolipids (SLs), comprising ceramides (Cer) and complex SLs, serve as an essential structural component of cell membranes and bioactive signaling molecules. An increasing body of published data demonstrates active remodeling of cellular SL metabolism during infection, highlighting this pathway as a promising antiviral target (Smith et al., 2022; H et al., 2024; Yager and Konan, 2019; Avota et al., 2021; Thomas et al., 2023). SLs modulate distinct stages of viral replication across diverse virus families. For instance, members of the *Flaviviridae* family, pathogens imposing a substantial global health burden, exploit various lipid species, including SLs for host cell entry, genome replication, particle formation, and egress (Evangelista et al., 2026). Specifically, sphingomyelin (SM) and glycosphingolipids are indispensable for hepatitis C virus (HCV) genome replication (Khan et al., 2014; Hirata et al., 2012). Different flaviviruses depend on the sphingolipid metabolic network at distinct life-cycle stages: Zika virus (ZIKV) requires it for particle assembly, dengue virus (DENV) for genome replication, and west Nile virus (WNV) for replication platform formation and replicon assembly (Leier et al., 2020; Konan et al., 2022; Wang et al., 2016; Martin-Acebes et al., 2014; Martin-Acebes et al., 2016). Similarly, the lipid envelope of HIV is highly enriched in SLs, which facilitate Siglec-1 mediated virus capture by macrophages and DCs (Hammonds et al., 2017; Puryear et al., 2012), while galactosylceramides contribute to HIV membrane fusion and entry (Fantini et al., 1997). Furthermore, SL biosynthesis is essential for norovirus internalization (Orchard et al., 2018), as well as for influenza virus entry and viral RNA synthesis (Tafesse et al., 2013; Jung et al., 2025). Emerging evidence also highlights a critical role for Cer in coronaviral replication (Salisch et al., 2025; Mitchell et al., 2026).

Our previous work demonstrated that plasma membrane ceramides, induced during MV glycoprotein contact with uninfected bystander T cells, play a central role in cytoskeleton paralysis and the inhibition of proliferation (Gassert et al., 2009). This established the immunosuppressive function of excessive ceramide accumulation triggered by contact with MV-infected cells. Additionally, we showed that DC-SIGN-activated acid sphingomyelinase (aSMase) is crucial for trafficking the MV entry receptor, CD150, to the cell surface, thereby facilitating MV entry into immature monocyte-derived dendritic cells (MoDCs) (Avota et al., 2011). Furthermore, elevations in Cer and sphingosine-1-phosphate (S1P) levels were observed in MV-infected BJAB cell line, and pharmacological inhibition of sphingosine kinase 1 (SK1) or acid ceramidase impaired MV replication in both: cell lines and human PBMCs (Vijayan et al., 2014; Grafen et al., 2019). Mechanistically, the SL inhibitors ceranib-2 and SKI-II suppress downstream mTORC1 signaling and viral protein synthesis in infected peripheral blood lymphocytes (PBLs) (Chithelen et al., 2022). More recently, S1P-mediated extrusion of MV-infected cells was detected in the apical wash of respiratory epithelial cells (Brockhurst et al., 2025). However, the precise impact of MV infection on SL dynamics within primary immune cells remains poorly characterized.

Our group and others have delineated the roles of Cer and neutral sphingomyelinase 2 (nSMase2) in controlling neutral lipid storage within lipid droplets (LDs), a specialized intracellular storage organelles. This functional link highlights a critical crosstalk between sphingolipid and fatty acid metabolic pathways (Schempp et al., 2024; Robles-Martinez et al., 2025). LDs store esterified fatty acids primarily as triacylglycerols (TGs) and are frequently hijacked by viruses. Viral pathogens exploit LDs either as platforms for replication and particle assembly or as metabolic reservoirs to provide lipids for membrane synthesis and mitochondria energy production via fatty acid β-oxidation (Farias et al., 2024). Well- characterized examples include HCV, SARS-CoV-2, Dengue virus, and Zika virus, all of which enhance LD biogenesis in close proximity to their replication organelles (ROs) to facilitate virus replication and particle morphogenesis (Herker, 2024; Farley et al., 2022; Miy et al., 2007; Eggert et al., 2014; Samsa et al., 2009; Dias et al., 2023). Conversely, growing evidence indicates that LD induction also serves as a hallmark of host innate immune responses during viral infection, underscoring a complex, dual role for these organelles (Monson et al., 2021a; Monson et al., 2018; Bosch et al., 2020; Hinson and Cresswell, 2009).

While host lipid metabolism in MV-infected primary human lymphocytes remains largely unexplored, pioneering studies in persistently infected BGM cells identified a significant increase in TGs (Anderton et al., 1983). Anderton *et al.* detected elevated levels of unsaturated fatty acids within released virions, suggesting these lipids influence virus particle properties (Anderton et al., 1982). Interestingly, the expression of genes involved in fatty acid synthesis were found to be downregulated 24 h post-infection (hpi) in COBL-a cells, a cell line established from human umbilical cord blood (Sato et al., 2008). Together, these findings highlight the need to further elucidate how MV manipulates host lipid profiles during acute infection.

A critical barrier in characterizing lipid metabolism in MV-infected cells has been the scarcity of data from primary cell models. To address this limitation, the current study utilizes human primary CD4^+^ T cells isolated from healthy donors for MV infection, followed by comprehensive lipidomic profiling. By integrating untargeted and targeted lipidomics, we identified three major lipid classes altered in MV-infected CD4^+^ T cells: SLs, TGs and glycerophospholipids. Remarkably, TGs and glycerophospholipids in infected cells exhibited a distinct shift towards long-chain, unsaturated species. Furthermore, SLs represented the lipid class as one of most significantly affected by MV infection in CD4^+^ T cells. Specifically, we observed a significant increase in dihydroceramides (dhCer) and Cer, coupled with a substantial decrease in hexosylceramides (HexCer) at 48 and 72 hpi. To dissect the functional roles of these specific lipid species in viral pathogenesis, pharmacological inhibitors targeting their respective metabolic pathways were deployed in CD4^+^CD150^+^ T cells. Our data suggest a critical role for glucosylceramide synthase (GCS) activity in MV glycoprotein-mediated cell-to-cell fusion, essential mechanism for viral entry and spread. Furthermore, we show that both TG metabolism and *de novo* sphingolipid synthesis are crucial for the production of infectious progeny in CD4^+^ T cells. In conclusion, this study establishes the pro-viral nature of elevated sphingolipid and TG levels in MV infection in primary human T cells.

## 2 Materials and methods

### 2.1 Ethics statement

Primary human cells from healthy blood were obtained through the blood donor program of the Department of Transfusion Medicine, University of Wuerzburg, and analyzed anonymously. All experiments involving human material were conducted according to the principles expressed in the Declaration of Helsinki and ethically approved by the Ethical Committee of the Medical Faculty of the University of Wuerzburg. Ethical approval statement number 2026-241-dvhd was issued by the ethics committee of the faculty of Medicine of University of Wuerzburg for the usage of anonymous biomaterials in the project within DFG funded Research Training Group 2581, RTG 2581-41785 7878.

### 2.2 Primary CD4^+^ T cell culture, viability assessment and CD150 expression analysis

Primary human peripheral blood mononuclear cells (PBMCs) were isolated from healthy donors by gradient centrifugation on Histopaque-1077 (Sigma-Aldrich, Germany). CD4^+^ T Cells were enriched (≥90%) using MagniSort™ Human CD4 T cell Enrichment Kit (Invitrogen by Thermo Fisher Scientific) and maintained in RPMI 1640/10% FCS. For stimulation, cells were incubated with 1 μg/mL α-CD3 (clone UCHT-1, BD Bioscience) and α-CD28 (clone CD28.2, BD Bioscience) for 20 min on ice, subsequently transferred to 96-well plates precoated with 25 μg/mL α-mouse IgG (Dianova) (2 h at 37 °C) and incubated at 37 °C for up to 3 days. Toxicity of inhibitor treatments was tested using AnnexinV Apoptosis Detection Kit (Biolegend). CD150 PE conjugate from Becton Dickinson (Cat.No. 559592) was used for the analysis of CD150 surface expression by flow cytometry.

### 2.3 Virus propagation, infection and quantification

The recombinant wild-type MV strain IC3223-EGFP (Hashimoto et al., 2002) was propagated on Vero cells expressing CD150 (Vero-SLAM) and utilized to infect anti-CD3/CD28 activated CD4^+^ T cells, which were either left untreated or pretreated with specific inhibitors. Total virus yield (comprising both cell-associated and supernatant virions) was harvested from the cell cultures via a single freeze-thaw cycle of the complete cell culture volume, followed by titration on Vero-SLAM cells.

### 2.4 Pharmacological inhibitors and reagents

All chemical inhibitors and agonists were sourced from commercial suppliers as follows. Sigma-Aldrich/Merck: inhibitors targeting diacylglycerol *O*-acyltransferases (DGAT), including the DGAT1 inhibitor A922500 and the DGAT2 inhibitor PF-06424439; sterol *O*-acyltransferase (SOAT) inhibitors, including the dual SOAT1/2 inhibitor avasimibe and the SOAT1 specific inhibitor K604; the carnitine palmitoyltransferase 1 (CPT1) inhibitor etomoxir; serine palmtoyltransferase (SPT) inhibitors myriocin and L-cycloserine; the ceramide synthase (CerS) inhibitor fumonisin B1; and the liver X receptor (LXR) agonist GW3965. Hycultec: the alternative dual SOAT1/2 inhibitor pactimibe. Biomol: glucosylceramide synthase (GCS) inhibitors, including the racemic mixture DL-threo-PDMP (Cat.No. Cay10005276) and its inactive enantiomer (-)-L-threo-PDMP (Cat.No. Cay1005278). The active inhibitory enantiomer, D-threo-PDMP, is supplied within the racemic DL-threo-PDMP mixture and is referred to as D-PDMP throughout this manuscript. MedChemExpress: the human adipose triglyceride lipase (ATGL)-specific inhibitor NG-497 (Cat.No. HY-148756). The concentrations of the inhibitors used in cell culture and the viability analysis of treated CD4^+^ T cells are provided in the Supplementary Material (Supplementary Figure S1). We utilized etomoxir at a concentration of 5 μM, as concentrations exceeding this threshold in human T-cell cultures leed to a loss of CPT1 specificity and inhibit glutamine oxidative metabolism, as previously reported (O'Connor et al., 2018).

### 2.5 Virus binding and viral glycoprotein-mediated cell-to-cell fusion assays

For the viral binding assay, primary CD4^+^ T cells were seeded in triplicates at a density of 1 × 105 cells per well in 96-well plates and were either left untreated or pretreated with specific inhibitors for 24 h. To allow viral attachment while preventing entry, cells were incubated with wild-type MV (Fleckenstein strain; MOI = 5) for 2 h on ice, followed by extensive washing steps to remove unbound virions. Cell attached virus particles were quantified via flow cytometry targeting the viral glycoprotein H with an in-house generated H-specific monoclonal antibody (NC32). To investigate MV glycoprotein H- and F-dependent membrane fusion independently of viral replication, a virus-free cell co-culture assay was developed. In this system, HEK293 effector cells stably expressing wild type MV F protein and tetracycline (Tet)-inducible H protein were co-cultured with CD150-expressing Vero-SLAM cells. Syncytium formation was triggered by overnight induction with 1 μM Tet in the presence or absence of specific lipid-modulating pharmacological inhibitors. Subsequently, the cells were fixed, permeabilized and stained with Phalloidin-ATTO565 (Sigma-Aldrich/Merck) and DAPI to visualize cell boundaries and nuclei, respectively. Confocal laser scanning microscopy imaging was performed using a LSM 510 Meta (Zeiss, Germany), equipped with an inverted Axiovert 200 microscope and a 40× or 63× EC Plan-Apo oil objective (numerical aperture 1.3 or 1.4, respectively) and laser lines 488, 514, 561 and 633 nm. Images were acquired using Zeiss LSM software 3.2 SP2. The resulting relative syncytial area in the Tet-treated cells was quantified using the open-source image processing software package Fiji.

### 2.6 Oleic acid treatment

Oleic Acid (OA) medium was prepared as described previously (Listenberger and Brown, 2007). Briefly, OA (Sigma-Aldrich/Merck) was dissolved in NaOH solution and complexed to fatty acid free BSA (Sigma-Aldrich/Merck) before it was added to RPMI 1640/10% FCS in the desired concentration. Primary human CD4^+^ T cells were either left untreated or loaded with oleate at 50 μM concentration during anti-CD3/CD28 stimulation for 72 h.

### 2.7 Thin-layer chromatography (TLC) based GCS assay

Activated primary CD4^+^ T cells (3 × 10^6^ cells) were either left untreated, infected with MV IC323-EGFP (MOI = 0.4), or treated with specific inhibitors. Following incubation for 24, 48 or 72 h, cells were disrupted by 4 freeze-thaw cycles in liquid nitrogen in GCS lysis buffer comprising 20 mM HEPES (pH 7.4), 10 mM β-glycerophosphate and a protease inhibitor cocktail. The enzymatic activity of glucosylceramide synthase (GCS) in resulting cell lysates was assessed as described previously (Li et al., 2023). Briefly, 40 μg of total cell lysate protein was incubated with 10 μM NBD-C6-Ceramide (N-[6-[(7-nitro-2-1,3-benzoxadiazol-4-yl)amino]hexanoyl]-D-erythro-sphingosine, Avanti Polar Lipids) and 2 mM UDP-glucose (Biomol, Cayman Chemical, Cay15602) in an enzyme assay buffer containing 50 mM Hepes (pH 7.4), 25 mM KCl, 5 mM MnCl_2_ for 30 min at 37 °C. Reactions were terminated by the addition of methanol:chloroform (2:1, v/v) and lipids were isolated via Bligh and Dyer lipid extraction (Bligh and Dyer, 1959). Dried lipid extracts were reconstituted, spotted onto a TLC plate (Macherey-Nagel), and developed using methanol:chloroform:H_2_O (25:65:4, v/v/v). Plates were air-dried, and the fluorescent lipid spots were visualized under blue light excitation at 470 nm. Quantification was performed using an Odyssey Fc Imaging System (LI-COR Biosciences).

### 2.8 Fluorescence-based sphingomyelinase activity assays

Neutral sphingomyelinase (nSMase) activity in primary CD4^+^ T cells was determined as previously described (Mueller et al., 2014; Tonnetti et al., 1999). The acid sphingomyelinase (aSMase) activity assay was performed according to the manufacturer’s protocol (Biosynth Carbosynth). Briefly, 1 × 10^6^ CD4^+^ T cells were either left uninfected or infected with MV IC323-EGFP for 72 h. Cells were subsequently disrupted by four freeze/thaw cycles at −80 °C in either 40 μL of detergent-free nSMase lysis buffer (20 mM Tris pH 7.4, 10 mM β-glycerophosphate, 5 mM DTT, protease inhibitors) or 40 μL of aSMase lysis buffer (250 mM sodium acetate (pH 5.2), 0.1% NP-40, 1.3 mM EDTA, protease inhibitors). Post nuclear supernatants (10 μL cell lysate in final volume of 30 μL) were incubated overnight at 37 °C with 1.35 mM HMU-PC (6-hexadecanoylamino-4-methylumbelliferyl-phosphorylcholine, Biosynth Carbosynth) dissolved in nSMase lysis buffer or aSMase lysis buffer with aSMase assay mixture supplemented with 1.3 mM EDTA. overnight. Fluorescence was measured at an excitation wavelength 404 nm and an emission wavelength of 460 nm. All enzymatic activity data were normalized to total protein content.

### 2.9 Cell fractionation and plasma membrane isolation

Primary CD4^+^ T cells (1 × 10^7^ cells) were stimulated in 6-well plates, and either were left untreated or treated with specific inhibitors for 24 h. Plasma membrane (PM) fractions were isolated using the Minute™ Plasma Membrane Protein Isolation and Cell Fractionation Kit (Invent Biotechnologies) according to the manufacturer’s instructions, with slight modifications. Briefly, instead of utilizing the provided filter cartridges, cells were disrupted by passing them through a 26-gauge needle after incubation in the kit’s lysis buffer. A subsequent centrifugation step at 1,600 rpm for 5 min at 4 °C was applied to pellet the nuclei from the cytosolic and PM fractions. The final PM pellets were immediately frozen and stored at −80 °C prior to lipid extraction.

### 2.10 Western blot analysis

Total cell lysates (15 µg of protein) were resolved by SDS-PAGE and transferred to membranes for immunoblotting. After incubation with specific primary and secondary antibodies, protein bands were visualized using SuperSignal West Pico PLUS detection reagent (Thermo Fisher Scientific) and quantified using LI-COR software (LI-COR Biosciences). The primary antibodies utilized were sourced as follows: the MV H-specific antibody was generated in-house (polyclonal rabbit serum, H45), and the GAPDH-specific antibody was purchased from Santa Cruz Biotechnology (sc-47724). Furthermore, antibodies targeting SPTLC1 (Cat. No. 15 376-1-AP) and SPTLC2 (Cat. No. 68094-1-Ig) were obtained from Proteintech, and the GCS-specific antibody (Cat. No. 12869-1-AP) was purchased from Thermo Fisher Scientific.

### 2.11 Quantification of lipid remodeling in MV-infected cells by UPLC-qTOF-MS

Lipid extraction and analysis of 1 × 10^6^ primary CD4^+^T cells (either left uninfected or infected for 72 h) were conducted using an ACQUITY UPLC system coupled to a Synapt G2 HDMS qTOF-MS (all from Waters), following a previously published protocol (Mueller et al., 2015). The sole modification was that the electrospray ionization (ESI) source was operated both in positive and negative modes. Triacylglycerol, cholesterol, cholesteryl ester, and glycerophospholipid species were identified by matching the retention times within the extracted ion chromatograms generated from their major positively charged ions. Quantification was achieved using integrated peak areas and an internal standard approach (response factor of one). Chromatogram acquisition, processing, peak detection, and integration were performed using MassLynx and QuanLynx (version 4.1; all from Waters).

### 2.12 Quantification of sphingolipids (canonical and deuterium-labeled) by liquid chromatography tandem-mass spectrometry (LC-MS/MS)

Cells were harvested, resuspended in 500 µL methanol and subjected to sphingolipid extraction as described (Prell et al., 2024). To this end, 1 mL methanol/chloroform (1:1, v:v) was added that contained the internal standards d_7_-dihydrosphingosine (d_7_-dhSph), d_7_-sphingosine (d_7_-Sph), 17:0 ceramide (d18:1/17:0), d_31_-16:0 sphingomyelin (d18:1/16:0-d_31_), 17:0 glucosyl(β) ceramide (d18:1/17:0) and 17:0 lactosyl(β) ceramide (d18:1/17:0) (all from Avanti Polar Lipids). Final extracts were subjected to LC-MS/MS sphingolipid quantification applying the multiple reaction monitoring (MRM) approach. Chromatographic separation was achieved on a 1290 Infinity II HPLC (Agilent Technologies) equipped with a Poroshell 120 EC-C8 column (3.0 × 150 mm, 2.7 µm; Agilent Technologies) guarded by a pre-column (3.0 × 5 mm, 2.7 µm) of identical material. MS/MS analyses were carried out using a 6495C triple-quadrupole mass spectrometer (Agilent Technologies) operating in the positive electrospray ionization mode (ESI+). Chromatographic conditions and settings of the ESI source and MS/MS detector have been published elsewhere (Salisch et al., 2025; Prell et al., 2024). Peak areas of Cer, dhCer, SM, dhSM, HexCer and LacCer subspecies, as determined with MassHunter software (version 10.1, Agilent Technologies), were normalized to those of their internal standards followed by quantification via external calibration. DhSph and Sph were directly quantified via their deuterated internal standards. After incubation of cells with d_3_-palmitate, *de novo* formed sphingolipids, which had a d_3_-label (sphingoid backbone) or a d_6_-label (additional labeling of the 16:0 side chain), were quantified using the calibration curves of the unlabeled analogues.

### 2.13 RNA extraction and RT-qPCR analysis

Anti-CD3/CD28 stimulated primary CD4^+^ T cells (1 × 10^6^ cells) were either left uninfected or infected with MV IC323-EGFP (MOI = 0.4) for 24, 48 or 72 h. Total RNA was isolated using the GenElute^MT^ Mammalian Total RNA Miniprep Kit (RTN350-1KT, Sigma-Aldrich/Merck) following the manufacturer’s instructions. One-step cDNA synthesis and real-time quantitative PCR (RT-q) amplifications were performed on a LightCycler 96 System (Roche) using the LightCycler Multiplex RNA Master kit (Roche, Cat. No. 07083173001). Primer sequences specific for the catalytic subunits of SPT complex are available on OriGene Technologies homepage (SPTLC1, Cat. No. HP209369, SPTLC2 Cat. No. HP208118). Primer sequences for the quantification of ORMDL1, ORMDL2, AND ORMDL3 mRNAs were synthesized as published previously (Lee et al., 2020). Dual labelled fluorescent probe sequences were designed using SnapGene Software (GSL Biotech). SPTLC1 PROBE: 5′-FAM-GGA GTC CCT TTC TCC AGC CTT TCA-BHQ1-3’; SPTLC2 PROBE: 5′-FAM-CAC AAC AAT ATG CAA AGC CTA GAG AAG-BHQ-3’; ORMDL1 PROBE: 5′-FAM-CTG GCA AGT TTC TAT ACG AAG TAT GAT CCA-BHQ1-3‘; ORMDL2 PROBE: 5’-FAM CCG AGT GAT GAA TAG CCG AGG CAT-BHQ1-3‘; ORMDL3 PROBE: 5’-FAM CAG TTC ACG GCC TCT CGG AAG TT BHQ-3‘. Target mRNA expression levels were normalized to RNase P as an internal reference control. HURNASEP FW: 5’- AGA TTT GGA CCT GCG AGC G-3’; HURNASEP RV: 5′-GAG CGG CTG TCT CCA CAA GT-3’; HURNASEP PROBE: 5′- FAM-TTC TGA CCT GAA GGC TCT GCG CG-BHQ1-3’.

### 2.14 Electron microscopy

Sample preparation for electron microscopy was conducted according to a previously published protocol (Prufert et al., 2004). Transmission electron microscopy was performed at 120 kV acceleration voltage at a JEM-1400 Flash (JEOL, Germany) transmission electron microscope equipped with a Matataki 2 k × 2 k camera.

### 2.15 Statistical analysis

Data were acquired in at least three independent experiments involving individual donors when primary human CD4^+^ T cells were used. Data were analyzed using GraphPad Prism software (GraphPad Software, Inc.). For comparing two groups, a two-tailed Student’s t-test was performed. When applicable, P-values were adjusted with Welch’s correction to account for unequal variances. For comparing more than two groups, One-way ANOVA or Two-way ANOVA were used. Dunnett’s post-hoc test was then performed for multiple comparisons against the respective control group. Where indicated, individual pairwise comparisons between groups were performed using two-tailed students t-tests. Statistical significance is indicated as follows: *p < 0.05, **p < 0.01, ***p < 0.001, ****p < 0.0001; and ns, not significant. P-values are shown as *p < 0.05, **p < 0.01, ***p < 0.001, and ***p < 0.0001; ns, non-significant).

## 3 Results

### 3.1 Distinct changes in the CD4^+^ T cell lipidome after MV infection: accumulation of ceramides and triacylglycerols with a decrease in glycerophospholipids

**FIGURE 1:**
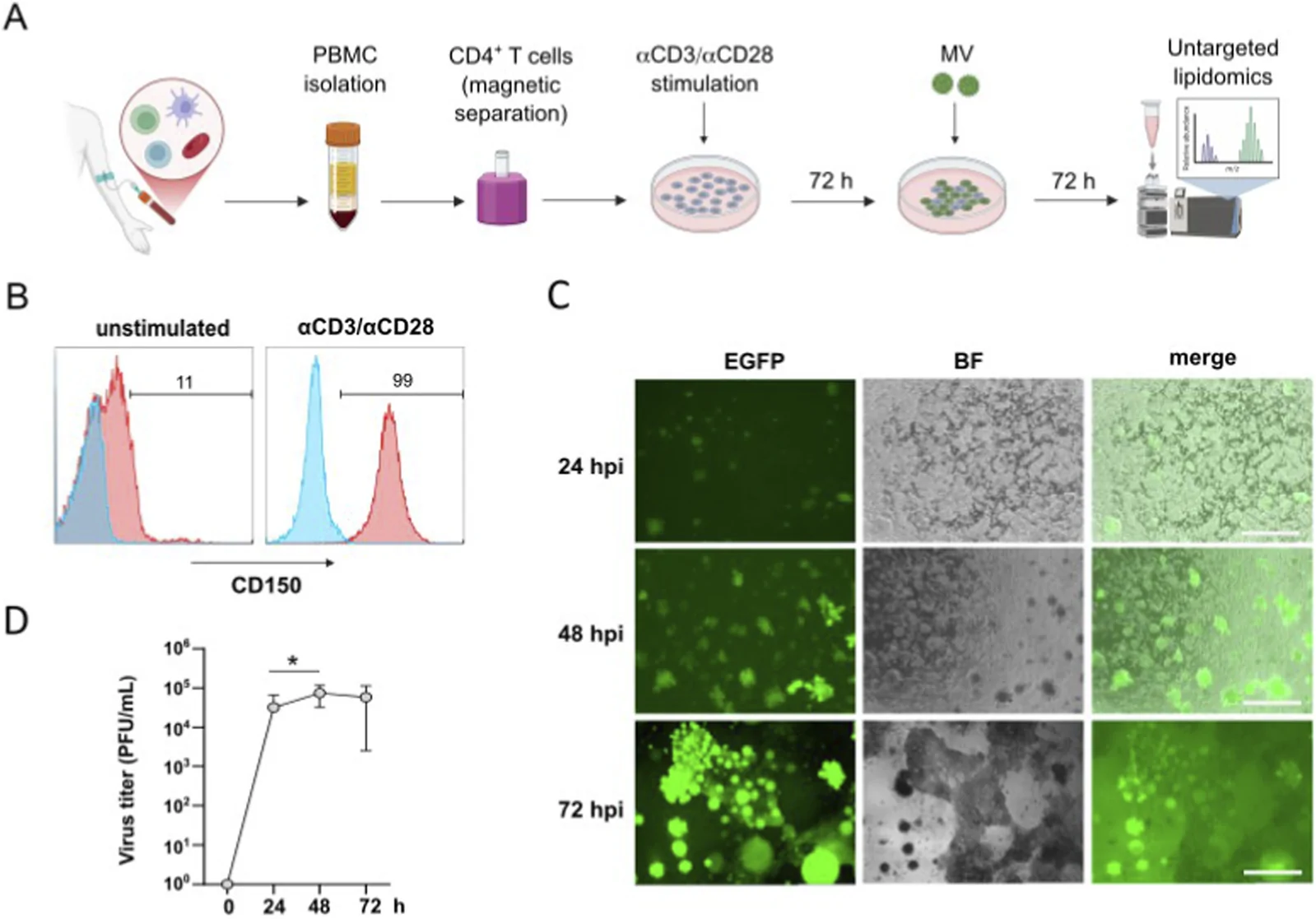
Sample preparation workflow and validation of MV infection in primary CD4^+^ T cells. **(A)** Experimental timeline detailing negative magnetic isolation of CD4^+^ T cells from human PBMCs, polyclonal activation via anti-CD3/CD28 for 3 days, and infection with the recombinant wild type MV strain IC323-EGFP (MOI = 0.4, 3 days) prior to lipid extraction and lipidomic analysis. Created with BioRender.com. **(B)** Representative flow cytometry histograms showing cell-surface upregulation of the MV entry receptor CD150 on anti-CD3/CD28 activated (72 h) versus unstimulated CD4^+^ T cells. **(C)** Viral replication kinetics monitored via EGFP fluorescence and **(D)** quantification of infectious virus production at 24, 48 and 72 hpi. Scale bar: 400 µm.

To investigate the impact of MV infection on the cellular lipidome, CD4^+^ T cells were isolated from healthy donor blood via negative selection (Figure 1A). The cells were stimulated with αCD3/αCD28 antibodies for 3 days to upregulate the surface expression of MV entry receptor CD150, which enables viral entry and dissemination (Figure 1B). Infection was performed using the recombinant wild-type MV-strain IC323-EGFP, allowing infection progression to be monitored via EGFP fluorescence. Concomitantly, extensive syncytia formation was observed in the infected cultures as the number of virus infected, EGFP positive cells increased (Figure 1C). Titration of viral yields from the infected cell cultures demonstrated that infectious progeny particles were detectable as early as 24 h post-infection (hpi). Viral titers increased significantly by 48 hpi and remained elevated for the subsequent 24 h (Figure 1D). Cells were harvested for lipid analysis 3 days post-infection. At this time point, the majority of cells expressed both EGFP and MV glycoprotein H, confirming successful viral entry and viral protein translation (Figure 1C; Supplementary Figure S2).

**FIGURE 2:**
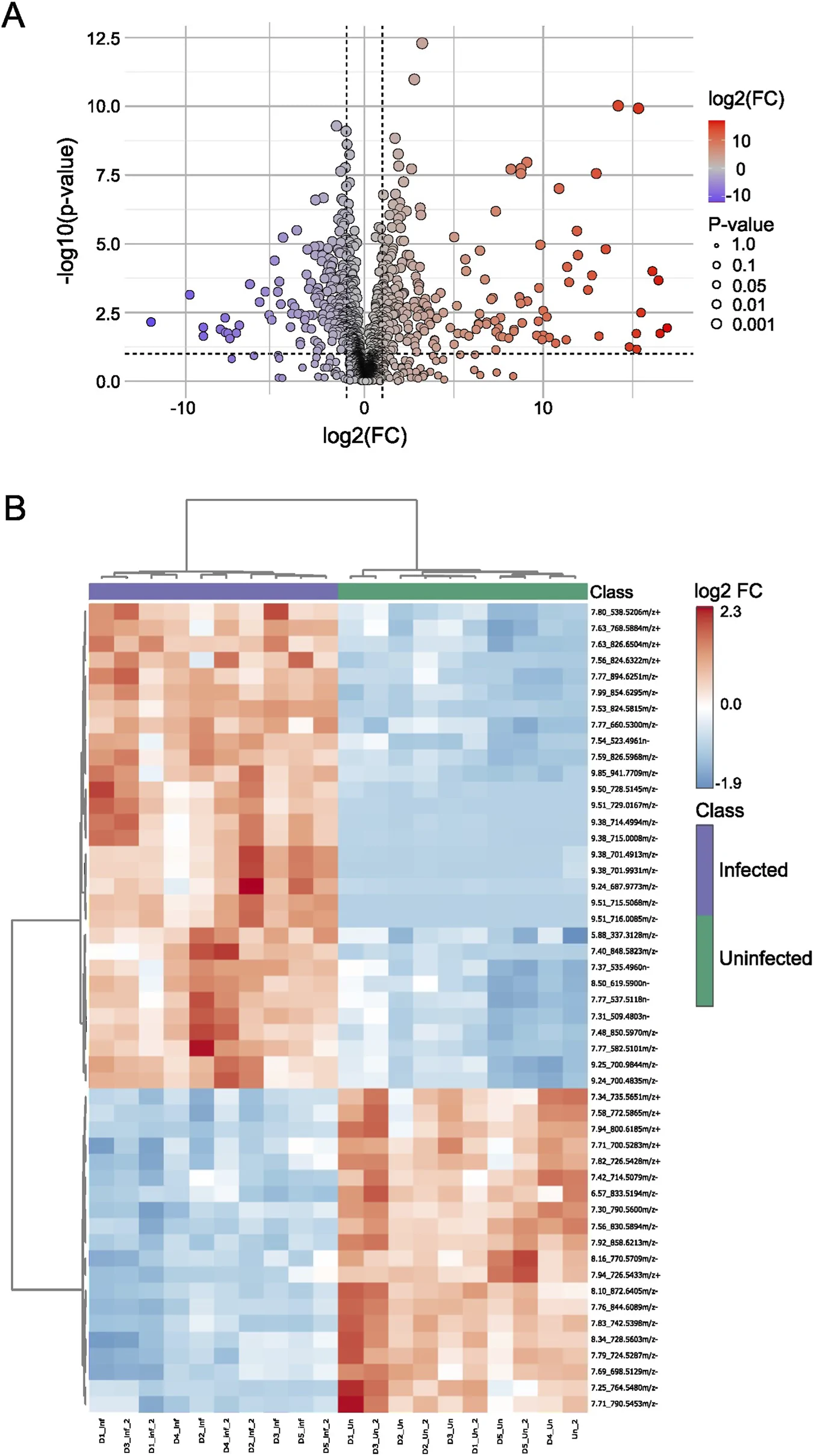
Measles virus infection drives global remodeling of the CD4^+^ T cell lipidome. Untargeted lipidomic profiles from 5 independent donors (two technical replicates per donor), analyzed across two separate days using UPLC-qTOF-MS. **(A)** Volcano plot illustrating the quantitative distribution of significantly regulated lipids following MV infection. The x-axis displays the log2-transformed fold change (FC) of lipid abundance in MV-infected versus uninfected control cells. The y-axis displays the -log10-transformed p-value derived from a Student’s test. Each data point represents a unique lipid feature. Lipids exhibiting a fold change greater than 4-fold change in either direction are highlighted in color, with the size of each point corresponding to its statistical significance. **(B)** Hierarchical clustering heatmap displaying the top 50 significantly altered lipid species identified by univariate analysis between infected and uninfected control groups (ANOVA, p-value <0.001, FDR <0.01%). Rows represent unidentified lipid features analysed via positive and negative electrospray ionization (ESI^+/−^), labelled by their specific retention time and mass-to-charge ratio (m/z). Columns represent individual blood donor samples. Dendrograms on the left and top represent hierarchical clustering trees for lipids and samples, respectively. Red indicates significant upregulation, while blue indicates significant downregulation.

**FIGURE 3:**
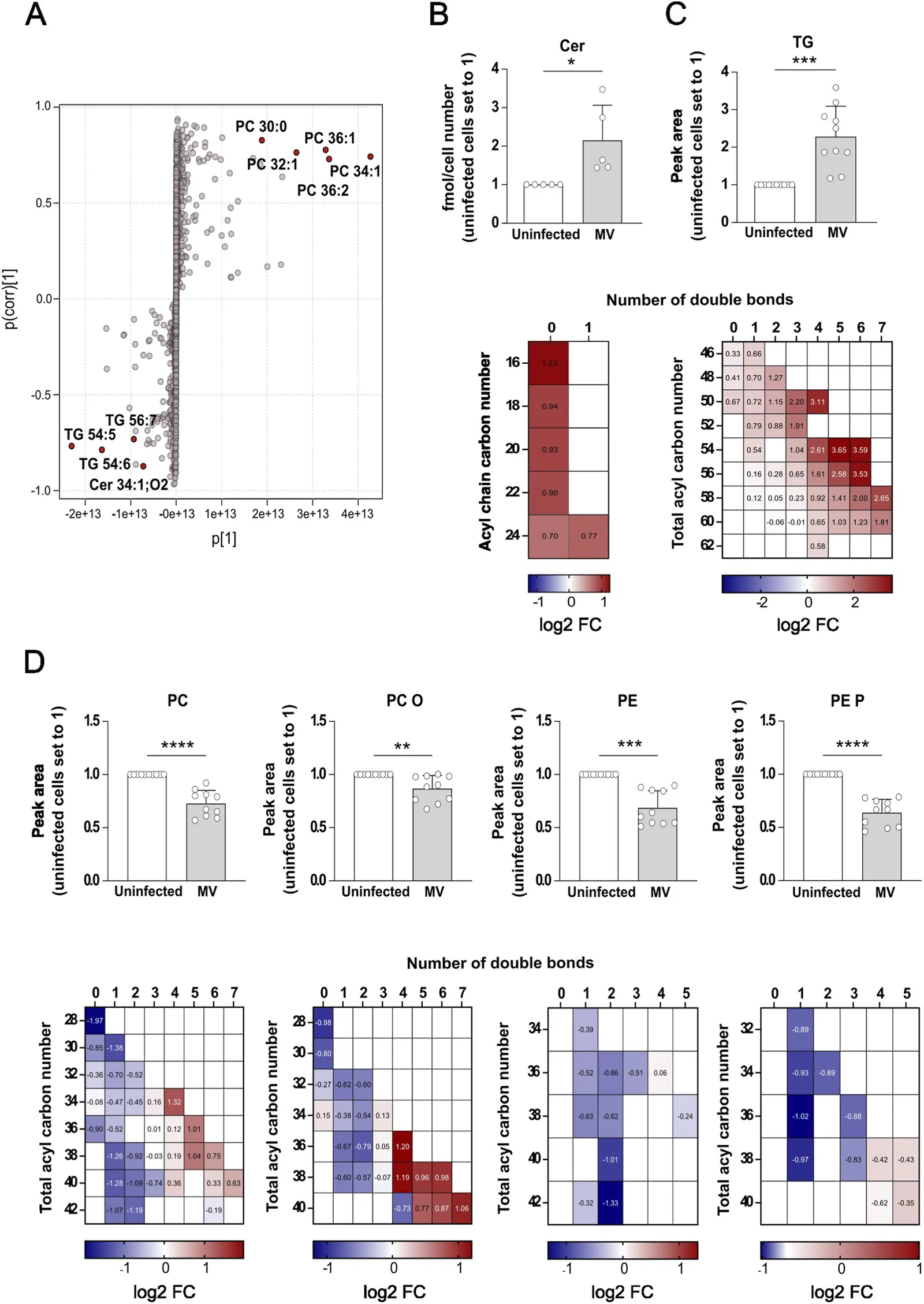
Ceramides, triacylglycerols and glycerophospholipids are the predominant altered lipids in MV-infected cells. **(A)** OPLS-DA S-plot illustrating the covariance [p (Allen et al., 2018), x-axis] versus correlation [p (corr) (Allen et al., 2018), y-axis] for all detected lipid species from untargeted lipidomics. Each data point represents an individual lipid feature. Lipids positioned in the upper-right and lower-left regions contribute most strongly and reliably to the discrimination between MV-infected and uninfected cohorts. Highlighted lipid labels were assigned using in-house developed data base. **(B–D)** Relative abundance and fold changes of **(B)** ceramides (Cer), **(C)** triacylglycerols (TG) and **(D)** glycerophospholipids. PC, phosphatidylcholine; PC O, ether-linked phosphatidylcholine; PE, phosphatidylethanolamine; PE P, plasmenylethanolamine. *Upper panels*: Relative abundance levels in infected versus uninfected controls. Data are presented as mean ± standard deviation (SD), with individual donor samples depicted as circles. *Lower panels*: Heatmaps displaying the log_2_ fold changes mapped by carbon number (rows) and number of double bonds (columns), with fold change values represented by the color scale. Statistical significance was determined using an unpaired, two-tailed Student’s t-test (* <0.05, ** <0.01, ***p < 0.001, ****p < 0.0001).

To determine whether MV infection induces changes in the cellular lipidome, lipid profiles from infected and uninfected cells were compared across five independent donors. Out of the 2,378 features detected, 404 lipid features (17%) were significantly different (p < 0.05, FC (log2) >2 or <2). Of these, 209 were higher and 195 were lower in infected cells than in uninfected cells, as visualized in volcano plot (Figure 2A). Hierarchical clustering analysis of the top 50 lipid features identified by analysis of variance (ANOVA) revealed that infected and uninfected samples naturally cluster separately based on their intensities (Figure 2B). Therefore, principal component analysia (PCA), was expected to discriminate between the two groups; however, the resulting scores did not show separation (Supplementary Figure S3A). The results indicate that the impact of MV infection on the lipidome of host cells is specific rather than being a dominant source of overall variability. While PCA, an unsupervised multivariate approach that captures the largest sources of variation in the data, did not separate the groups (Supplementary Figure S3A), ANOVA, which is supervised univariate statistical method, detected significant differences in lipid features between infected and non-infected cells (Figure 2). We therefore used Orthogonal Partial Least Squares-Discriminant Analysis (OPLS-DA), a supervised multivariate approach, to identify the statistically significant features that distinguish between uninfected and infected samples. The score plot (Supplementary Figure S3B) clearly separated the two groups, indicating the model’s discriminatory performance (R2Y = 0.984, Q2 = 0.943). Model robustness and statistical validity were further confirmed by a 1000-permutation test, which yielded significant results for both R^2^Y and Q^2^ (p < 0.001). The S-Plot (Figure 3A) identified a small subset of variables with high covariance and correlation values that were primarily responsible for the separation between the groups. By matching the mass-to-charge ratios and retention times of the lipid features with high covariance with those in the in-house developed database, one ceramide (Cer; Cer 34:1; O2), three triacylglycerols (TG; TG 54:4, TG 56:7, TG 54:6) and five glycerophosphocholines (PC; PC34:1, PC36:1, PC36:2, PC 32:1, PC 30:0) were identified (Supplementary Table S1).

Next, the levels of all detectable lipid species of Cer, TG and selected glycerophospholipids, such as PC, ether-linked phosphatidylcholine (PC-O), phosphatidylethanolamine (PE) and plasmenylethanolamine (PE-P), were analyzed in the samples. The levels of the identified lipids within each lipid class were summed (Figures 3B–D, upper panels), and log2 fold changes, calculated as the ratio of averages between infected and non-infected cells, were then visualized in a heat map (Figures 3B–D, lower panels). The results revealed that total levels of Cer and TGs were twice as high (Figures 3B,C, upper graphs), while total levels of PC, PC O, PE and PE P were slightly but significantly lower (Figure 3D, upper graphs) in MV-infected CD4^+^ T cells compared to their uninfected controls. Strikingly, MV infection also altered the molecular composition of TGs and glycerophospholipids: the relative proportion of long-chain polyunsaturated fatty acids (PUFAs) was enhanced within the TG, PC, PC O and PE P lipid pools (Figures 3C,D, lower panels).

Summarizing, sphingolipids (Cer), storage lipids (TGs), and membrane lipids (glycerophospholipids) emerged as the most affected classes, showing the highest magnitude of change consistently across all donors. Because glycerophospholipids are the most abundant and primary structural components of cell membranes, these alterations likely affect physical membrane properties.

### 3.2 Differential regulation of ceramide, dihydroceramide and hexosylceramide levels in MV- infected cells

**FIGURE 4:**
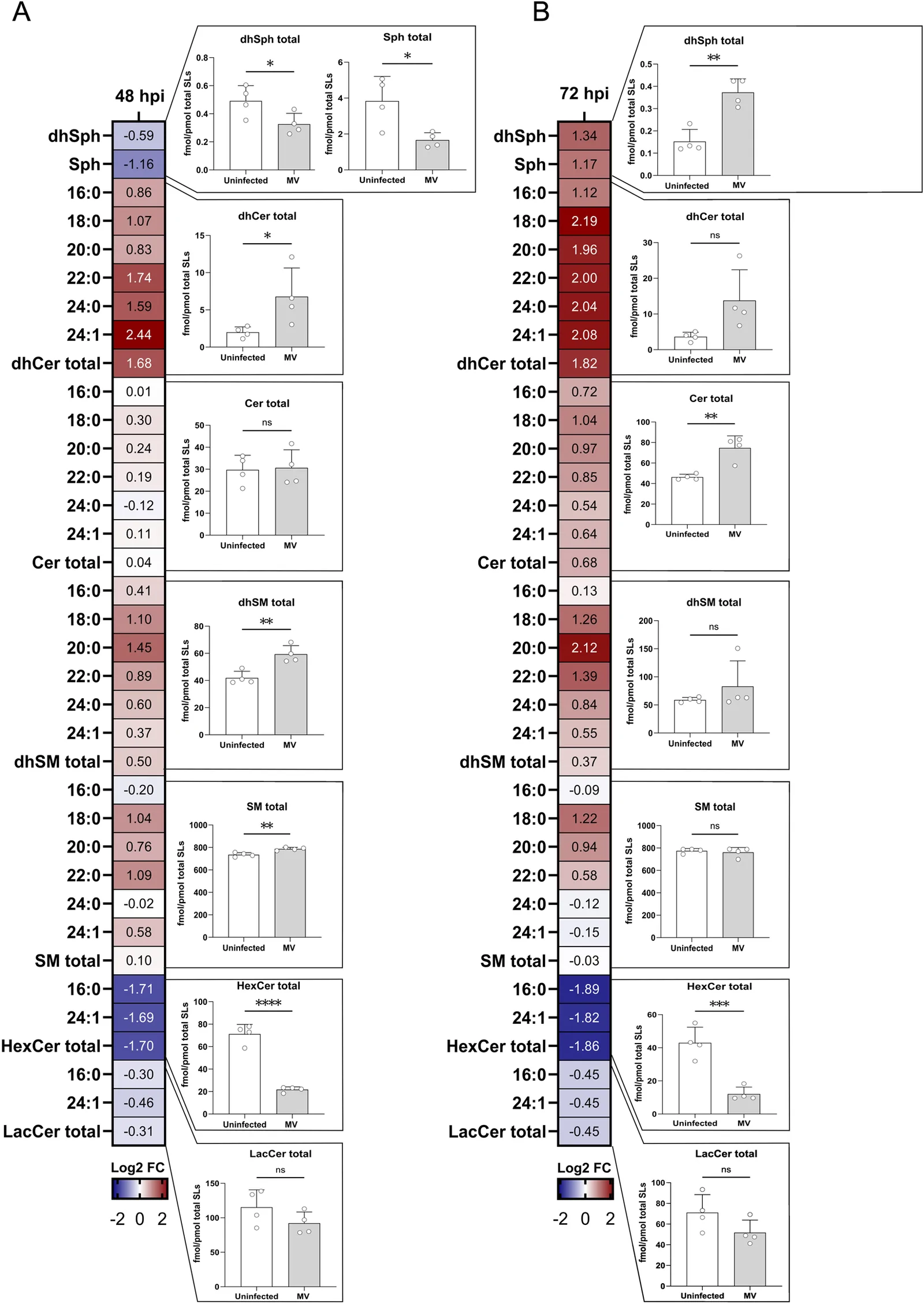
Sphingolipid levels are profoundly dysregulated in MV-infected CD4^+^ T cells. **(A)** Relative changes in specific sphingolipid (SL) species were determined by LC-MS/MS in primary CD4^+^ T cells at **(A)** 48 hpi and **(B)** 72 hpi with MV IC323-EGFP compared to uninfected control cells (n = 4). *Heatmaps*: Rows represent log_2_ fold-change values of individual SL species in infected versus uninfected cells. *Bar graphs*: Summary of changes across all monitored metabolites within each sphingolipid class relative to the total pool of SLs (fmol/pmol total SLs). Data are presented as mean ± SD (relative to uninfected control) with individual donor samples represented by circles. Statistical significance compared to uninfected control (white bar) was determined using an unpaired, two-tailed Student’s t-test (* <0.05, ** <0.01, ***p < 0.001). dhSph, dihydrosphingosine; Sph, sphingosine; Cer, ceramide; dhCer, dihydroceramide; dhSM, dihydrosphingomyelin; SM, sphingomyelin; HexCer, hexosylceramide; LacCer, lactosylceramide.

It is well documented that MV replication is positively affected by the activity of acid sphingomyelinase in monocyte-derived dendritic cells, and by the activities of acid ceramidase and sphingosine kinases in peripheral blood lymphocytes (Avota et al., 2011; Grafen et al., 2019). A plethora of published data demonstrates the remodeling of SL signatures in virus-infected cells (Thomas et al., 2023; Dai et al., 2024). However, it remains unclear whether and to what extend MV infections alters the SL landscape in primary lymphocytes. Fingerprinting analysis from our untargeted lipidomics approach revealed an upregulation of ceramides (Figures 3A,B). Specifically, lipid profiling identified six distinct ceramide species whose levels were higher in infected cells compared to the control samples (Figure 3B, lower panel). To obtain a more comprehensive view of the sphingolipid landscape, we performed a targeted lipidomic analysis. CD4^+^ T cells isolated from the peripheral blood of eight donors were either left uninfected or infected for 48 or 72 h prior to lipid extraction and subsequent sphingolipid quantification using a highly sensitive LC-MS/MS-based method (Figure 4).

The results of the targeted analysis confirmed a significant upregulation of Cer at 72 h post-infection (hpi) (Figure 4B, third panel). In addition, the increase in dihydroceramide (dhCer) levels were even higher than those of Cer, albeit showing donor dependent variations (Figure 4B, second panel). In line with elevated dhCer levels, dihydrosphingosine (dhSph), the metabolic precursor for dhCer synthesis, was significantly upregulated in infected cells (Figure 4B, upper panel). In contrast, hexosylceramides (HexCer) were significantly and consistently downregulated in all donors following infection (Figure 4B, second panel from the bottom). Although total levels of sphingomyelin (SM) and dihydrosphingomyelin (dhSM) did not change significantly, specific SM species, namely, SM(d18:1/18:0), SM(d18:1/20:0), and SM(d18:1/22:0), were higher in infected cells (Figure 4B, third panel from the bottom). Given that sphingomyelin synthases lack intrinsic selectivity for fatty acid chain length, these data may indicate a selective activation of ceramide synthases CerS1 and CerS4.

At 72 hpi, large adherent syncytia exhibited reduced GFP fluorescence intensity compared to single infected cells or smaller clusters in suspension (Figure 1C). This reduction suggests a potential shutdown of host cell protein synthesis during the late stages of infection (Tiwarekar et al., 2018; Inoue et al., 2009; Sato et al., 2007). To avoid confounding effects from fusion-induced cellular stress, sphingolipid levels in infected cells were analyzed at an earlier time point (48 hpi, Figure 4A). The dysregulation pattern of dhCer and HexCer levels was highly consistent between 48 hpi (Figure 4A) and 72 hpi (Figure 4B). Importantly, Cer levels increased significantly only at 72 hpi, indicating a possible late-stage stress response. Interestingly, dhSph and Sph exhibited opposite trends between the two time points; both lipids accumulated at 72 h post-infection following an initial decrease at 48 h compared to controls. The accumulation of dhCer and dhSM at 48 hpi suggests a metabolic bottleneck along the *de novo* sphingolipid synthesis pathway. The accumulation of dhSM is likely a compensatory mechanism driven by sphingomyelin synthase (SMS) activity, which converts excess of dhCer into dhSM.

### 3.3 MV infection suppresses *de novo* sphingolipid synthesis and glucosylceramide synthase activity

**FIGURE 5:**
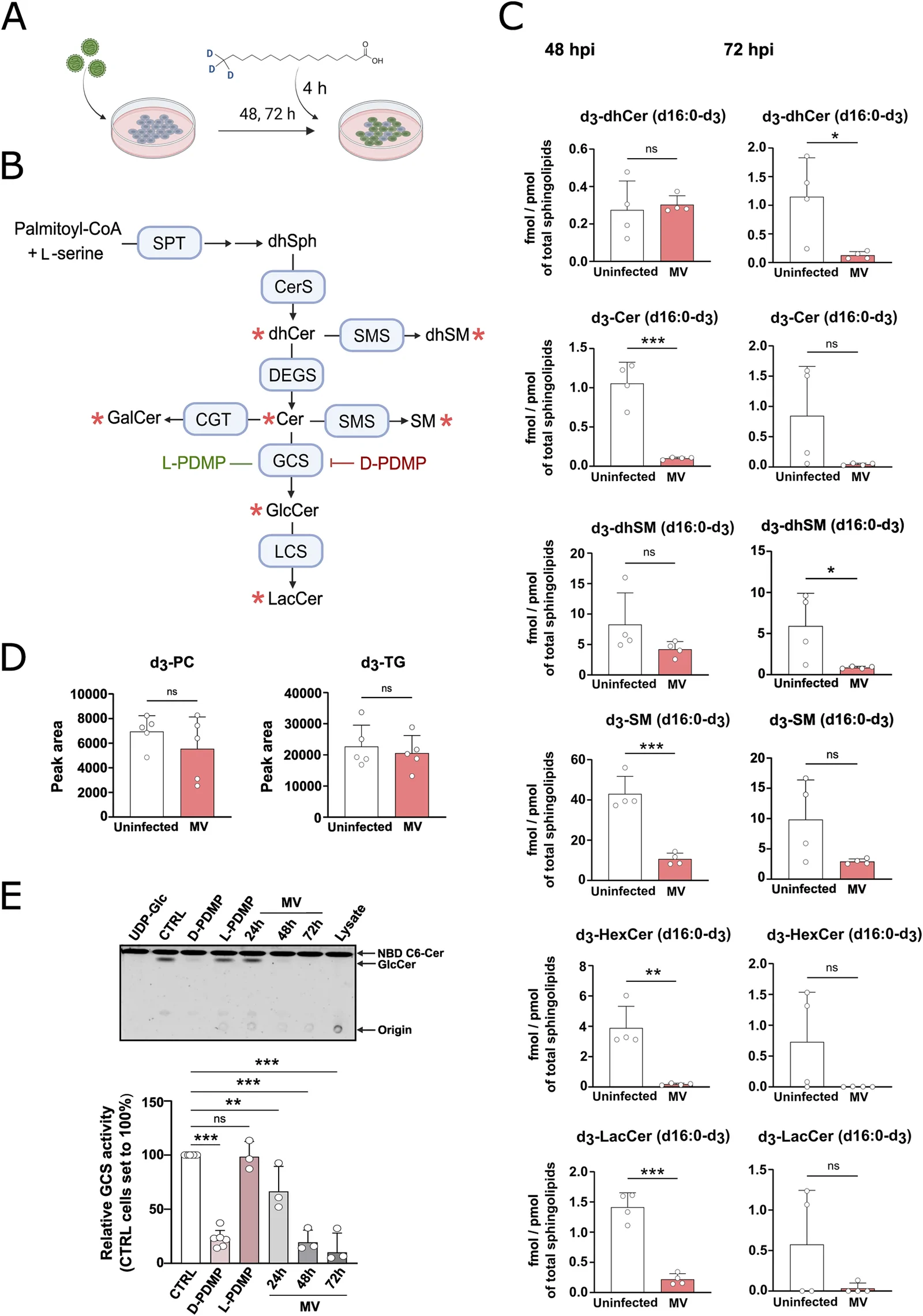
MV infection inhibits the *de novo* sphingolipid synthesis pathway **(A)** Experimental schema of the metabolic *de novo* lipid synthesis assay. Infected and uninfected CD4^+^ T cell cultures were pulsed with 10 µM [*d*_*3*_]-C16 palmitate during the final 4 h of incubation prior to lipid extraction and LC-MS/MS analysis. Created by BioRender.com. **(B)** Overview of selected steps and enzymes within the sphingolipid *de novo* synthesis pathway relevant to this study. Deuterated sphingolipid metabolites monitored by LC-MS/MS are indicated with an asterisk (*). Pharmacological modulators of GCS activity: the inhibitor (D-PDMP) and stimulator (L-PDMP), are highlighted. **(C)** Relative abundance of deuterated metabolites measured at 48 hpi (left columns) and 72 h hpi (right columns). Dual incorporation of [*d*_*3*_]-C16 palmitate yielding [*d*_*6*_]-C16 sphingolipids is integrated into the data analysis. Bar graphs illustrate mean ± SD from four independent donors. It should be noted that, under the chromatographic conditions used, GalCer and GlcCer cannot be separated and were determined as a sum as HexCer. **(D)** Incorporation of [*d*_*3*_]-C16 palmitate PC (left graph) and TG (right graph) extracted from uninfected and infected CD4^+^ T cells at 72 hpi, quantified by UPLC-qTOF-MS (n = 5) **(E)** Representative thin-layer chromatography (TLC) image (upper panel) and summarized quantification (lower panel) of GCS enzymatic activity assays in cell lysates from MV-infected cells at 24, 48 and 72 hpi (n = 3) utilizing NBD C6-ceramide as a substrate and showing NBD C6-GlcCer (“GlcCer”) as the product. The GCS inhibitor D-PDMP served as a negative control. Statistical significance compared to uninfected controls (white bars) was evaluated using an unpaired, two-tailed Student’s t-test (* <0.05, ** <0.01, ***p < 0.001). SPT, serin-palmytoyltransferase; CerS, ceramide synthase; SMS, sphingomyelin synthase; DEGS, dihydroceramide desaturase; CGT, UDP-glucose ceramide galactosyltransferase; GCS, UDP-glucose ceramide glucosyltransferase; LCS, lactosylceramide synthase.

The accumulation of dhSM species likely reflects an enhanced flux through the *de novo* sphingolipid synthesis pathway. Crucially, dhSM is synthesized upstream of DEGS activity, which introduces the characteristic 4,5-*trans* double bond into the dhCer sphingoid base. To investigate whether enhanced *de novo* sphingolipid synthesis drives the accumulation of dhCer and Cer in MV-infected CD4^+^ T cells, we performed a *de novo* synthesis assay at 48 and 72 hpi. Stable isotope-labelled [*d*_*3*_]-C16 palmitate was added to the culture medium of both uninfected and infected cells during the final 4 h of incubation, followed by LC-MS/MS analysis (Figure 5A). Unexpectedly, quantification of the [*d*_*3*_ */d*_*6*_]-labelled sphingolipid (SL) species revealed a significant decrease in labelled products downstream of dhCer within the *de novo* pathway at 48 hpi (Figures 5B,C, left column). By 72 hpi, labeled fractions were barely detectable or completely absent in all measured SL species from infected samples (Figure 5C, right column). These findings suggest that MV infection initially impairs dihydroceramide desaturase (DEGS) activity at 48 hpi, resulting in an increased [*d*_*3*_ */d*_*6*_]-dhCer-to-[*d*_*3*_ */d*_*6*_]-Cer ratio. Subsequently, *de novo* dhCer production stalls by 72 hpi, resulting in minimal label incorporation across all detected SL pools. Conversely [*d*_*3*_ */d*_*6*_]-palmitate labeling of PC and TG pools remained comparable between uninfected and MV-infected cells at 72 hpi, indicating that palmitate uptake and availability for general lipid synthesis were undisturbed (Figure 5D). This suggests a metabolic shift characterized by a redirection from *de novo* sphingolipid synthesis toward TG and PC biosynthesis in MV-infected cells.

The initial, rate-limiting step in *de novo* sphingolipid synthesis is the condensation of L-serine and palmitoyl-CoA by the serine palmitoyltransferase (SPT) complex to yield 3-ketodihydrosphingosine. Transcriptional analysis of the SPT complex subunits revealed a significant reduction in mRNAs specific for the catalytic subunits SPTLC1 and SPTLC2, as well as the regulatory subunit ORMDL1, in infected cells at 72 hpi (Supplementary Figures S4A,B). Western blot analysis validated this downregulation, showing reduced SPTLC1 and SPTLC2 protein levels at 72 hpi (Supplementary Figure S4C). However, at this late stage of infection, GAPDH protein levels were also diminished compared to 48 hpi time point, reflecting a global shutdown of host cell protein translation.

Despite the impairment of *de novo* biosynthesis, Cer levels still were higher in infected cells (Figure 4B), indicating that compensatory pathways are engaged. One plausible mechanism is the catabolism of the membrane lipids to counteract diminished SPT activity. However, contrary to this hypothesis, measurements of sphingomyelinase activity in cell lysates revealed that both acid (aSMase) and neutral (nSMase) sphingomyelinases exhibited reduced activity (Supplementary Figure S5). This indicates that the observed ceramide accumulation is not driven by sphingomyelinase activation. Ceramides also serve as precursors for complex sphingolipids, the deregulated metabolism of which can lead to ceramide buildup (Hussain et al., 2012). We observed a significant downregulation of HexCer (sum of glucosyl- and galactosylceramides) in MV-infected cells (Figure 4). This suggests either diminished activity of enzymes synthesizing HexCer from Cer, or elevated activity of cerebrosidases degrading HexCer back to ceramides; both scenarios would facilitated cellular ceramide accumulation. In T cells, GlcCer is the predominant monohexosylceramide (Reza et al., 2021). While expression levels of glucosylceramide synthase GCS remained comparable between uninfected and infected cells (Supplementary Figure S6), its enzymatic activity significantly decreased at 48 and 72 hpi (Figure 5E). The identification of this metabolic bottleneck provides a rationale for the paradoxical accumulation of ceramides despite a stalled *de novo* pathway.

### 3.4 Neutral lipids and *de novo* sphingolipid synthesis promote the generation of infectious measles virus progeny

**FIGURE 6:**
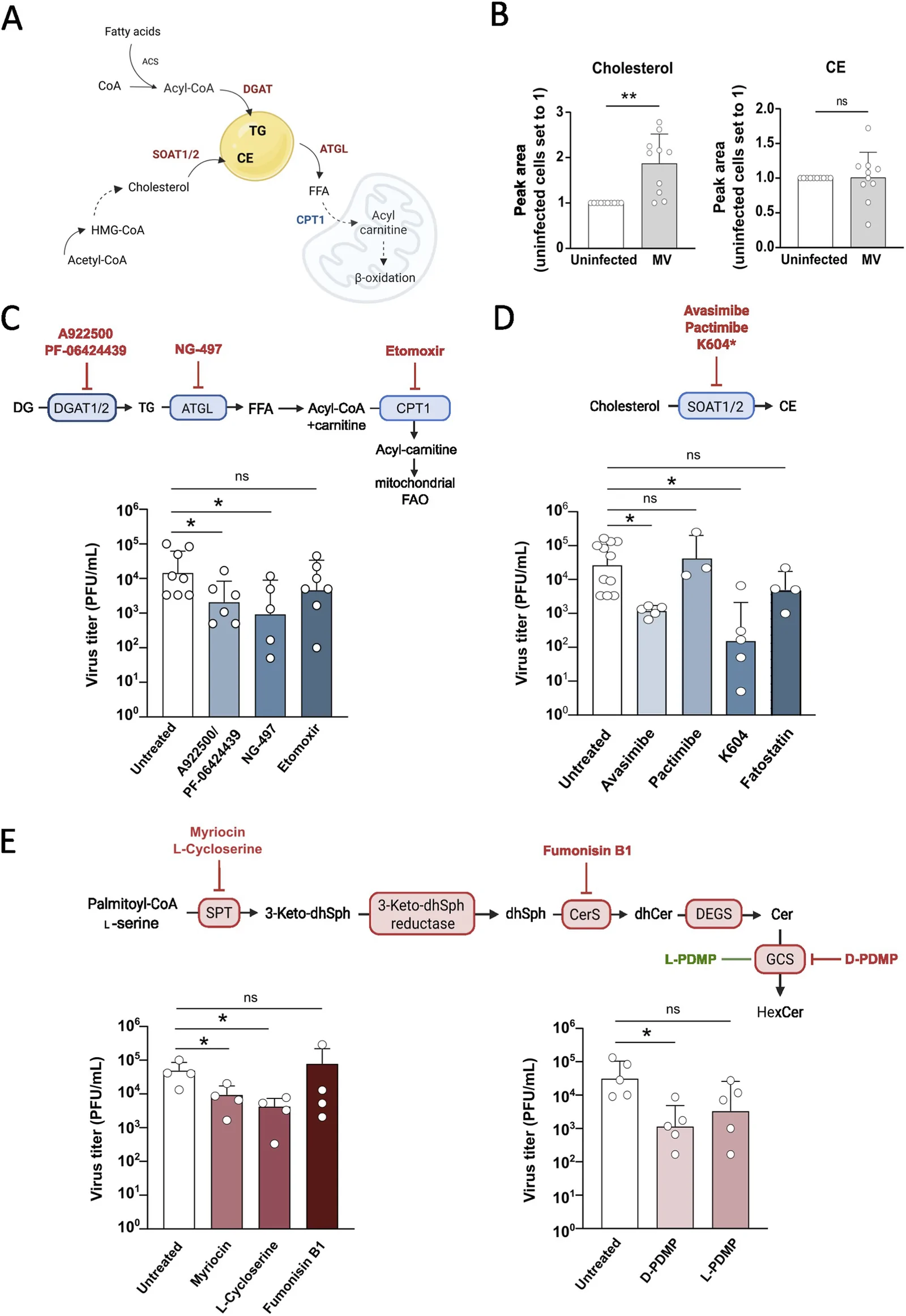
Neutral lipids and sphingolipids support MV replication **(A)** Simplified schematic representation of TG and cholesteryl ester (CE) synthesis, lipid droplet biogenesis, and neutral lipid mobilization. Lipolysis is initiated by adipose triglyceride lipase (ATGL) to release free fatty acids (FFA) for mitochondrial fatty acid β-oxidation. ACS, acyl-CoA synthetase; SOAT, sterol-*O*-acyltransferase; DGAT, diacylglycerol *O*-acyltransferase; CPT1, carnitine palmitoyltransferase 1. Created with BioRender.com. **(B)** Relative fold changes of cholesterol (left) and CE (right) in MV-infected cells at 72 hpi. Uninfected controls are set to 1. Data are presented as mean ± standard deviation (SD) from two technical replicates, each performed using CD4^+^ T cells isolated from five independent donors with individual analysed samples depicted as circles. **(C–E)** Primary CD4^+^ T cells were pretreated for 2 h with pharmacological inhibitors targeting **(C)** TG synthesis and mobilization **(D)** cholesterol and CE synthesis and **(E)**, sphingolipid *de novo* synthesis (left panel) or the glucosylceramide synthesis (right panel) prior to MV infection (MOI = 0.4). The SOAT1 specific inhibitor K604 is indicated by an asterisk (*). Viral titers were assessed at 48 hpi via plaque assay and expressed as plaque-forming units per ml (PFU/mL), determined by microscopic enumeration. Bar graphs represent mean ± SD, with individual biological replicates indicated by circles within the graphs. *Upper panels* show the schematic overviews of the metabolic pathways and specific enzymes targeted by the respective inhibitors. Statistical significance compared to uninfected controls (white bars) was evaluated using an unpaired, two-tailed Student’s t-test (* <0.05, ** <0.01, ***p < 0.001).

Excess of intracellular fatty acids and cholesterol are converted into neutral lipids: specifically, TG and cholesteryl esters (CE), which are stored in lipid droplets (LDs) and can be released as free fatty acids (FFA) via ATGL-dependent lipolysis (Figure 6A). We observed significantly higher TG levels in MV infected CD4^+^ T cells (Figure 3C), suggesting enhanced storage to meet cellular demand for energy and lipid building blocks during infection. In contrast to TG, levels of detectable CE in MV infected cells remained comparable to uninfected controls (Figure 6B, right panel). However, the overall cholesterol level was higher in infected cells than in the control group, albeit with notable donor-to-donor variability (Figure 6B, left panel).

While TG storage is most prominent in adipose and liver tissues, most cell types, including T cells, retain the capacity to form LDs when supplemented with fatty acids (Walther et al., 2017). To investigate the role of TGs in MV replication, we treated CD4^+^ T cells with the monounsaturated fatty acid oleic acid (OA) to promote TG accumulation. Following a 3-day infection period, infectious virus production was compared between OA-treated and untreated cells. In 13 out of 18 donors, OA treatment increased virus titers. Notably, this enhancement was more pronounced in T cells isolated during spring and summer months (Supplementary Figure S7A). In contrast, during late autumn and winter, only a few isolates showed a similar increase (Supplementary Figure S7B).

We next examined whether TG homeostasis is critical for MV replication. Pharmacological inhibition of the diglyceride acyltransferases DGAT1 and DGAT2, using the small-molecule inhibitors A922500 and PF-06424,439 respectively, resulted in a significant reduction of infectious virus production (Figure 6C). Similarly, interfering with the first step of lipolysis using the human ATGL-specific inhibitor NG-497 negatively affected infectious MV titers. However, blocking carnitine palmitoyltransfrerase 1 (CPT1) activity with etomoxir, thereby preventing fatty acid transport into the mitochondria for β-oxidation, did not significantly affect virus production (Figure 6C). These data suggest that the increase in TGs in MV-infected CD4^+^ T cells is proviral, with the storage and subsequent release of fatty acids from LDs promoting viral replication.

Several studies underline the importance of cholesterol in MV assembly, syncytia formation, and virus particle release (Manie et al., 2000; Vincent et al., 2000; Malvoisin and Wild, 1990). The synthesis of CE, the storage form of cholesterol, regulates the flux of cholesterol from the plasma membrane to intracellular compartments. We next investigated whether blocking CE synthesis, and subsequent increase of cholesterol in cellular membranes, would affect MV replication. To test this, the cells were treated with pharmacological inhibitors of sterol-O-acyltransferases (SOAT1 and SOAT2) prior infection. Specifically, we used the dual SOAT1/2 inhibitors avasimibe and pactimibe, as well as the SOAT1-selective inhibitor K604. Interestingly, both avasimibe and K604 significantly reduced the production of infectious virus, whereas pactimibe had no impact. Additionally, fatostatin was used to inhibit cholesterol synthesis, but it resulted in only slight, non-significant reduction in MV titers (Figure 6D). While these data suggest a proviral role for CE synthesis, the differential effects of the three SOAT inhibitors rise questions regarding the specific molecular targets essential for preventing MV replication.

Our lipidomics reveals a robust regulation of SLs during MV infection in CD4^+^ T cells, with dhCer and Cer showing significant upregulation (Figure 4). To investigate this further, we utilized myriocin and L-cycloserine to inhibit serine palmitoyltransferase (SPT), which catalyzes the rate-limiting step of *de novo* sphingolipid synthesis: the condensation of L-serine and palmitoyl-CoA to form 3-ketodihydrosphingosine (Figure 6E, scheme). Inhibition of this *de novo* pathway resulted in a moderate but significant decrease in MV titers. In contrast, the ceramide synthase inhibitor fumonisin B1, which acts two steps downstream of SPT, had no significant effect on MV replication (Figure 6E, bottom left). This suggests that MV may rely on metabolites upstream of ceramide synthase: 3-ketodihydrosphingosine or dihydrosphingosine, or one of their derivatives dihydrosphingosine 1-phosphate (dhS1P), which is known to trigger signaling pathways similar to those of S1P (Figure 6E, scheme).

Low levels of HexCer correlated with impaired GCS enzymatic activity in MV infected cells (Figures 4, 5E). To investigate the role of HexCer in MV infection, we treated CD4^+^ T cells with the GCS-modulating inhibitors L-PDMP and D-PDMP prior infection (Figure 6E, scheme). Both inhibitors reduced infectious virus titers; however, this effect was only significant for D-PDMP treated cells. In the case of L-PDMP, donor-dependent variations were more pronounced, and the reduction did not reach statistical significance (Figure 6E, right panel). Notably, GFP fluorescence, an indicator of the number of infected cells, was nearly absent in D-PDMP- treated cells. In contrast, fluorescence levels in L-PDMP-treated cells remained comparable to untreated controls (Supplementary Figure S8A). It is important to note that all inhibitors were used in concentrations which did not affect cell viability or surface expression of the MV entry receptor CD150 (Supplementary Figure S1).

### 3.5 Glucosylceramide synthase (GCS) activity is essential for MV attachment and glycoprotein mediated fusion during host cell entry

We observed that inhibiting GCS, DGAT, ATGL, SOAT or SPT negatively impacts the production of infectious viral progeny in CD4^+^ T cell cultures (Figures 6C–E). Using the recombinant wild-type MV strain IC323-EGFP, which carries the EGFP gene upstream of the N gene, allows for visualization of MV-infected cells via fluorescence microscopy. However, only a few inhibitory compounds reduced EGFP fluorescence and syncytia formation. Specifically, the emergence of EGFP fluorescence and cytopathic effects was blocked by combined pretreatment with DGAT1and DGAT2 inhibitors A922500 and PF-06424,439, as well as by single treatments with either the SOAT1/2 inhibitor avasimibe or the GCS inhibitor D-PDMP (Supplementary Figure S8). This suggests an inhibition of early steps of MV life cycle preceding viral replication and spread.

**FIGURE 7:**
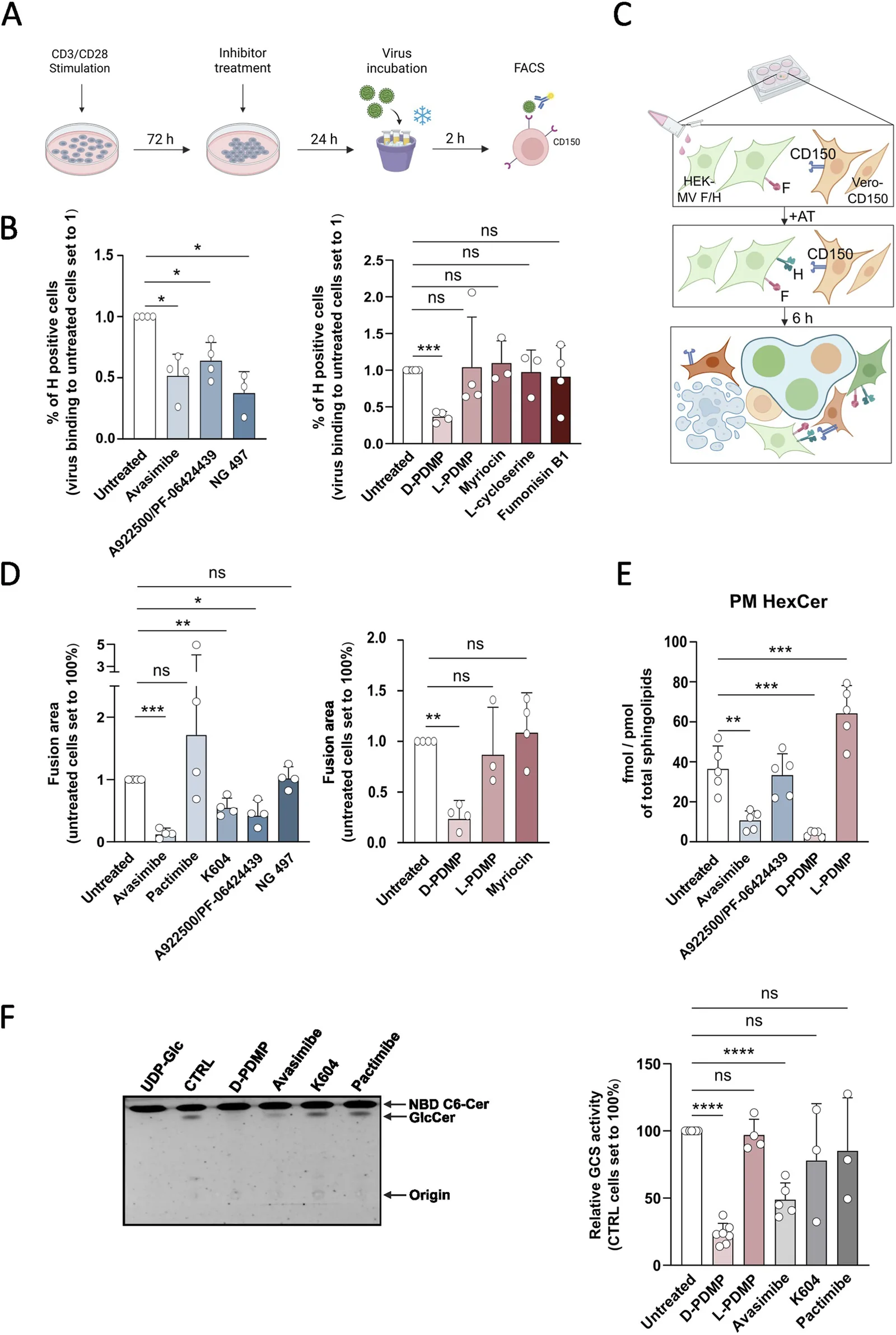
Triacylglycerol and glucosylceramide synthesis pathways are crucial for MV binding and glycoprotein-mediated cell-to-cell fusion. **(A)** Workflow for the virus binding assay. CD4^+^ T cells were stimulated with anti-CD3/CD28 antibodies for 72h, pretreated with specific pharmacological inhibitors for 24h, and subsequently incubated on ice with wild type MV (MOI = 5) for 2 h prior to flow cytometric analysis of MV H- protein positive cells. Created with BioRender.com. **(B)** Quantification of virus binding by flow cytometry following pretreatment with inhibitors targeting neutral lipid (left graph) or sphingolipid (right graph) homeostasis. **(C)** Schematic overview of the MV glycoprotein F- and H- mediated cell-to cell fusion assay. Vero cells stably expressing CD150 were co-cultured with HEK cells stably expressing MV glycoprotein F and inducible H. Co-cultures were pretreated with inhibitors overnight, followed by induction of MV H-protein expression with anhydrotetracycline (AT) for 6 h. Cells were subsequently fixed, permeabilized, and subjected to immunofluorescence staining of cytoskeleton and nuclei (Supplementary Figure S7). Syncytia formation was microscopically identified by the presence of multinucleated giant cells. **(D)** Relative fold-change in fusion area size in inhibitor-treated cells compared to untreated cells (set to 1), categorized by the lipid class targeted. Data for the neutral lipid inhibitors are shown on the left, and sphingolipid inhibitors are on the right. **(E)** LC-MS/MS quantification of HexCer in plasma membrane (PM) fractions isolated from CD4^+^ T cells left untreated or treated with inhibitors targeting SOAT1/2 (avasimibe), DGAT1 and DGAT2 (A922500 and PF-06424439) or GCS (D-PDMP, L-PDMP). **(F)** Representative thin-layer chromatography (TLC) image (left panel) and summarized densitometric analysis of NBD C6-GlcCer (“GlcCer”) bands in TLC images of GCS enzymatic activity assays in cell lysates utilizing NBD C6-ceramide as a substrate. The GCS inhibitor D-PDMP served as a negative control. Data represent mean ± SD, with circles indicating independent experiments. Statistical analyses were performed using an unpaired, two-tailed Student’s t-test with Welch’s correction (B, a paired, two-tailed Student’s t-test **(D,F)**, or a one-way ANOVA followed by Dunnett’s multiple comparison test **(E)**. Significance levels: p < 0.05 (*), p < 0.01 (**), p < 0.001 (***), p < 0.0001 (****).

Next, we investigated whether the inhibitors that reduced EGFP fluorescence intensity also affected virus attachment efficiency. Anti-CD3/CD28 stimulated CD4^+^ T-cells were pretreated overnight with the respective inhibitors and subsequently incubated with MV on ice to allow viral attachment while preventing entry. After extensive washing to remove unbound virus, the percentage of MV glycoprotein H-positive cells was quantified by flow cytometry (Figure 7A). Notably, avasimibe, the combined DGAT1/2 inhibitors (A922500/PF-06424439), NG-497 and D-PDMP significantly reduced the number of H-positive cells (Figure 7B), which directly correlates with the decreased EGFP fluorescence intensity observed in these infected cultures (Supplementary Figure S8). Interestingly, we observed increased viral attachment to oleate-treated cells (Supplementary Figure S7C). However, this enhancement varied markedly among CD4^+^ T cells isolated from different donors.

We developed a virus-free co-culture assay to investigate the effect of inhibitors on MV glycoprotein H- and F- dependent fusion. In this system, HEK cells stably expressing the wild type MV F protein and a tetracycline (Tet)-inducible H protein are co-cultured with CD150-expressing Vero cells. Fusion is triggered by Tet addition, and syncytia formation in inhibitor treated cultures is compared to that in untreated control cells (Figure 7C; Supplementary Figure S9). Interestingly, while blocking SPT activity with myriocin significantly reduced the production of infectious virus, it did not affect viral binding (Figure 7B, right panel), glycoprotein-mediated cell-to-cell fusion (Figure 7D, right panel) or GFP fluorescence in infected cultures (Supplementary Figure S8A). This suggests a positive role for the *de novo* sphingolipid pathway in post-entry virus replication. In contrast, inhibition of SOAT1/2 with avasimibe and GCS with D-PDMP resulted in an almost complete loss of fusion areas, whereas DGAT inhibition led to only partial reduction and OA treatment did not affect the fusion area (Figure 7D; Supplementary Figures S7D, S10). Notably, the efficiency of SOAT inhibition in preventing MV glycoprotein-mediated fusion varied substantially depending on the specific inhibitor used. While avasimibe was the highly effective, the alternative SOAT1/2 inhibitor pactimibe unexpectedly increased the fusion areas, whereas the SOAT1 inhibitor K604 exerted only a partial effect (Figure 7D). These divergent results point toward potential off-target effects of these compounds in cell culture and raise questions regarding the genuine molecular target required for MV glycoprotein mediated cell-to-cell fusion.

Mechanistically, the lipid composition of plasma membrane (PM) regulates both protein clustering on the cell surface and the physical parameters of membrane. To investigate the impact of these inhibitors on PM lipid composition, we performed a lipid analysis of isolated PM fractions from inhibitor-treated cells. We confirmed that combined treatment with DGAT1-and DGAT2-specific inhibitors reduced TAG levels in PM, while SOAT inhibition with avasimibe decreased CE content (Supplementary Figure S11A). Unexpectedly, SL analysis of PM fractions isolated from avasimibe-treated cells revealed a sharp reduction in HexCer and a significant upregulation of Cer levels (Figure 7E; Supplementary Figure S11B). This reduction of HexCer in the PM of avasimibe-treated cells was comparable to that induced by the GCS inhibitor D-PDMP. Furthermore, the overall SL profiles of avasimibe- and D-PDMP-treated cell membranes were nearly identical, suggesting that avasimibe may also act as a GCS inhibitor leading to decreased HexCer/Cer ratio in PM of inhibitor treated cells (Supplementary Figure S11C). To test this, we compared GCS activity in cell lysates treated with the three SOAT inhibitors to those treated with GCS inhibitor D-PDMP. Both avasimibe and D-PDMP significantly reduced GCS activity across all CD4^+^ T cell donors. Conversely, GCS sensitivity to K604 varied among donors, while pactimibe treatment showed no effect (Figure 7F). Because avasimibe and D-PDMP most efficiently prevented MV glycoprotein-mediated fusion initiation, we hypothesized that GCS activity is essential for this process. Although we cannot rule out a negative effect of avasimibe-induced CE downregulation on membrane fusion, our findings highlight a crucial role for GCS activity in the initial steps of viral entry.

## 4 Discussion

Positive strand RNA viruses reorganize host cell membranes to generate membranous viral factories for genome replication. Published data show the remodeling of glycerophospholipid, specifically PC, metabolism during orthoflavivirus (DENV, ZIKV, WNV), coronavirus, HCV and brome mosaic virus (BMV), infections (Hehner et al., 2024; Zhang et al., 2016; Muller et al., 2018; Sarwar and Randall, 2025). While negative-sense RNA viruses typically do not form membranous replication compartments, they use host lipids to anchor the proteins and assemble viral replication machinery in lipid enriched membraneless replication organelles, viral inclusion bodies. For many negative strand RNA viruses glycerophospholipids are important for triggering liquid-liquid phase separation during formation of viral inclusion bodies, virus assembly and budding (Chan et al., 2010). Already in 1977 a biochemical concept of cellular production of paramyxoviruses was postulated where the neutral lipid and phospholipid composition of PM is essential for the virus production (St Geme et al., 1977). More recently it was shown that MV and another paramyxovirus, NIPV, rely on PS and PI(4,5)P_2_ for virus assembly (Norris et al., 2022). The study from 1980 used an attenuated MV strain to infect follicular lymphoma cells showing increased enzyme activity in PC synthesis pathway and the incorporation of oleic acid in PC pool (Suzuki et al., 1980). Now we show the decrease in cellular PC levels at the late stage of infection which probably reflects the enhanced usage of the lipid for the formation of the virus envelop or reflects the need of cells to downregulate the membrane production as the total membrane surface area decrease upon virus-glycoprotein induced cell-to-cell fusion. Alternatively, the elevated levels of SM and dhSM species in MV-infected cells (Figure 4) may explain the reduced levels of PC (Figure 3D), as PC is consumed by sphingomyelin synthases (SMS). Furthermore, the accumulation of short and medium-chain saturated SM and dhSM species likely reflects an increased utilization of corresponding PC species during SM synthesis. This is further supported by the relative increase in long chain unsaturated species within the total PC pool (Figure 3D). Overall, this shift to long-chain unsaturated glycerophosphate species suggests the qualitative remodeling of the lipids which can contribute to the changes of plasma membrane mechanical properties to support virus budding. However, lowering the levels of long-chain polyunsaturated fatty acids (LC-PUFAs) by the inhibition of fatty acid desaturase 2 (FADS2) did not exert the inhibitory impact on virus replication in CD4^+^ T cells (data not shown) which suggests that shift to long-chain polyunsaturated glycerophospholipids is probably dispensable for virus replication.

Several lipidomics studies have observed the incorporation of oleic acid into cytosolic TGs in MV- infected cells, suggesting, but not directly demonstrating, lipid droplet accumulation (Anderton et al., 1982; Malvoisin and Wild, 1990; Suzuki et al., 1980; Anderton et al., 1983). Monson et al. showed that inducing lipid droplets prior to HSV-1 and ZIKV infection enhances the cellular production of type-I interferon (IFN). This primes the cells into an antiviral state, thereby decreasing viral replication (Monson et al., 2021b). In contrast, we observed higher MV replication in αCD3/αCD28 stimulated CD4^+^ T cells following LD induction via oleate treatment. This argues against an anti-viral role of lipid droplets in our specific experimental setup. Instead, we demonstrate that TG production and the ATGL-mediated first step of lipolysis are crucial for MV entry, specifically by supporting virus attachment to and fusion with target cells (Figures 6B,C; Supplementary Figure S7). Taken together, our data suggest that the TG pool exerts a positive impact on PM properties, thereby facilitating virus entry. In immune cells, LD formation can be induced by bacteria engagement primarily via TLR2 and TLR4 (Gopalakrishnan and Salgame, 2016). Because we have shown previously that MV can activate TLR2 signaling (Bieback et al., 2002) and now we show that MV infection increases cellular TG levels, we performed electronic microscopy analysis on CD4^+^ T cells at 24hpi. This revealed an increase in LDs compared to uninfected cells (Supplementary Figure S12). A previous study established that the DGAT2/ACSL5/CerS1 complex generates and sequesters acylceramides within LDs to mitigate ceramide toxicity and prevent cell death (Senkal et al., 2017). Given that we detect strong ceramide accumulation during MV infection, it is plausible that the LDs accumulating during MV infection contain acylceramides alongside TGs. This mechanism could protect cells from ceramide-induced toxicity, thereby extending the lifespan of infected cells until virus progeny is successfully produced.

Our lipidomic data show significant remodeling of sphingolipid content in MV infected CD4^+^ T cells (Figure 4). The most prominent changes are within dhCer and HexCer pools 48 hpi which get even more pronounced 72 hpi when most of measured sphingolipids including dhSph, Sph, dhCer, Cer, dhSM, SM are upregulated while precursors for complex sphingolipid synthesis, HexCer and LacCer, are strongly decreased. Opposite to expected enhancement, the gradual shutdown of *de novo* SL synthesis was caused by MV infection. 48 hpi low levels of incorporated [*d*_*3*_]-C16 labelled palmitate was detected in newly synthesized sphingolipids downstream of DEGS activity whereas 72 hpi all detected sphingolipids including dhCer were mostly negative for the palmitate label which was mainly used for the synthesis of PC and TAG species indicating the initial block in DEGS activity and redirection of lipid synthesis from sphingolipids to neutral lipid and glycerophospholipid synthesis.

Mechanistically, MV infection significantly reduced GCS activity as early as 24 hpi reaching nearly complete inhibition 72 hpi thereby indicating GCS as an early target in infection (Figure 5E). The impaired enzymatic activity of GCS, the main gate keeper for the synthesis of complex sphingolipids (including gangliosides) suggests the changes of PM lipid content and mechanical properties. *Cis*-Golgi and at less extent ER associate GCS catalyzes the transfer of glucose from UDP-glucose to ceramide to produce GlcCer. Concurrently, UDP-Glucose provides the sugar substrate for glycosylation and folding of viral glycoproteins important for virus replication and spreading while reducing UDP-glucose accessibility for GCS (Alarcon et al., 1988; Hodes et al., 1975). Loss of GCS activity in infected cells potentially cause increase of the GCS substrate Cer thereby creating the bottleneck at ceramide turnover in complex sphingolipids. The rise of Cer levels in infected cells is higher in comparison to the relatively stable SM content. Therefore, impaired activity of Cer transport protein (CERT) cannot be excluded as that would keep Cer in ER thereby making Cer unavailable for the sphingomyelin and HexCer synthesis in Golgi.

We cannot exclude that cellular stress response to virus infection negatively affects DEGS and GCS activity and/or localization in ER and Golgi respectively leading to accumulation of dhCer and Cer. Increased levels of SLs while shutting down new synthesis also points to the oxidative stress in infected cells. Recent study identifies DEGS and CERT as SL metabolic targets of p53, the master transcription factor that regulates cellular stress responses (Ghandour et al., 2026). It was shown that MV V protein binds p53 and prevents induction of subset of genes related to apoptosis, however, do not fully block p53 transcriptional activity (Cruz et al., 2006). Under stress conditions CerS1 can translocate from ER to Golgi and outer mitochondrial membrane (OMM) thereby bypassing classical *de novo* synthesis pathway to acetylate sphingosine and alter the local pool of SM (Sridevi et al., 2010; Oleinik et al., 2019). This could be the alternative way of SM and Cer synthesis in MV infected cells where we measured strongly decreased sphingolipid *de novo* synthesis pathway activity (Figure 5). However, the role of MV induced cellular stress as the driving force in the modulation of SL metabolism to promote viral replication is not resolved in this study and stays beyond the scope of this manuscript.

It was proposed that increased ceramides during orthoflavivirus infection correlates with the cytopathic effect and cell death in infected cell culture (Hehner et al., 2024). Ceramides play a critical role in coronaviral replication, with infection triggering ceramide accumulation via the sphingomyelinase pathway (Salisch et al., 2025; Mitchell et al., 2026). Our data indicate that MV is well adapted to utilizing elevated levels of Cer, dhCer and Sph in infected cells to support its replication. Despite stalled *de novo* synthesis, sphingolipids accumulate to high levels during MV infection. Unlike MV infection, which blocks *de novo* synthesis yet increases Cer content, serine palmitoyltransferase (SPT) inhibitor myriocin reduces overall sphingolipid levels in T cells (Blank et al., 2005). Importantly, viral progeny production is significantly impaired in myriocin treated cells, suggesting that the accumulation of sphingolipids in MV- infected cells serves a pro-viral function (Figure 6E, left panel). Our data support the hypothesis that MV infection induced accumulation of sphingolipids supports viral replication at post-entry stage as myriocin treatment do not affect virus binding and glycoprotein-mediated cell-to-cell fusion (Figures 7B,D, right panels).

We detected a significant downregulation of GCS activity and HexCer levels in MV-infected cells, alongside strongly elevated dhCer and Cer levels in infected CD4^+^ T cells 48 and 72 hpi. However, our virus-binding and MV glycoprotein-mediated cell-to-cell fusion assays demonstrate that GCS enzymatic activity is mandatory for efficient viral entry. This suggests that while GCS activity is not required to produce infectious viral progeny, it supports viral entry into host cells. Pharmacological inhibition of GCS prior to virus inoculation or before initiating the fusion assay, using inhibitor D-PDMP, strongly reduced virus attachment, as well as fusion between an MV H/F expressing cell line and MV receptor CD150 expressing cell line. Future work is required to fully elucidate the exact mechanism by which GCS activity regulates virus entry. Our data do not pinpoint PM HexCer levels as the primary factor governing virus attachment and fusion-mediated entry. Instead, the elevated Cer/HexCer ratio observed in D-PDMP treated cells may represent a critical driving factor. Interestingly, recently published data demonstrate that GCS inhibition alters the N-glycosylation of surface proteins (Pan et al., 2026). While we show that D-PDMP treatment of CD4^+^ T cells does not alter CD150 surface expression, we cannot exclude the possibility of changes in receptor clustering due to a modified lipid environment or altered glycosylation within the extracellular domain of CD150.

In addition to D-PDMP, avasimibe emerged as one of the most potent inhibitors, effectively preventing MV binding, fusion, and viral replication in CD4^+^ T cells. Avasimibe is mainly known as cholesterol metabolism-regulating compound that inhibits SOAT1 and SOAT2, thereby blocking cholesterol esterification. However, treatment with other SOAT pathway inhibitors, such as pactimibe and K604, exhibited differential effects on viral replication (Figure 6D) and MV glycoprotein-mediated fusion (Figure 7D). Unexpectedly, lipidomic analysis of inhibitor-treated cells revealed that both D-PDMP and avasimibe negatively affected HexCer levels in the PM fractions (Figure 7E; Supplementary Figure S11C). Subsequent enzymatic activity assays confirmed that only D-PDMP and avasimibe exerted an inhibitory effect on GCS, whereas pactimibe and K604 did not (Figure 6F). Taken together, the capacity of avasimibe and D-PDMP to suppress GCS activity correlates with their ability to restrict MV replication in CD4^+^ T cells.

Avasimibe is also known as a potent activator of pregnane X receptor (hPXR) which is shown to be hijacked by DENV to reprogram lipid metabolism and suppress the immune responses, but we did not address the impact of avasimibe on PXR activation in T cells here (Bautista-Olivier et al., 2025). Our data add a new aspect of metabolic regulation elucidated by avasimibe and underline the importance of the assessment of the off-target effects of pharmacological drugs to characterize the genuine therapeutic targets in anti-viral treatments. Further work performing genetic ablation of potential anti-viral targets is necessary to dissect the importance of lipid metabolites hypothesized in this study as important for viral replication in CD150^+^CD4^+^ T cells.

Our study reveals several key lipid metabolism modules affected during MV infection of activated CD4^+^ T cells. However, certain limitations constrain the hypothesis about the mechanisms and functional meaning of lipid metabolism modulation during MV infection. First, our experimental setup lacks lipid analysis at early time-points post-infection. Due to the low percentage of initially infected cells, even at high multiplicities of infection, bulk lipidomics cannot be reliably applied within the first minutes or hours. Second, the asynchronous nature of viral spread in cell culture introduces heterogeneity, making single cell approaches highly challenging. Third, although this *in vitro* CD4^+^ T-cell model effectively characterizes lipid remodeling in infected cultures, these data require validation in experimental settings that model the complex *in vivo* environment.

**FIGURE 8:**
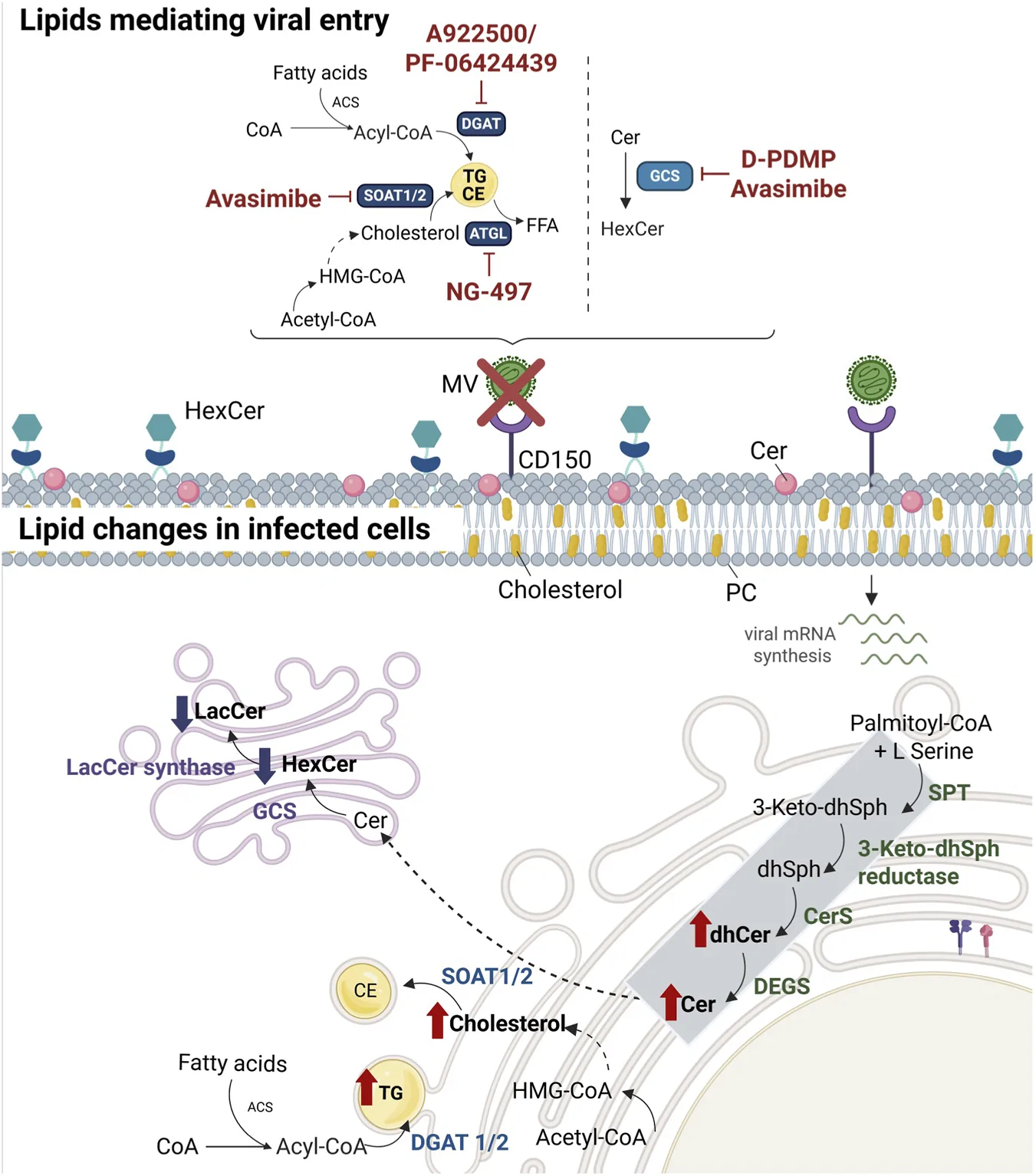
Summary of measles virus infection-mediated lipid remodeling and the functional roles of sphingolipids and neutral lipids in MV entry and replication in primary CD4^+^T cells. (Upper part) Inhibitors impairing virus binding and viral glycoprotein-mediated membrane fusion are depicted in red. Enzymes regulating neutral lipid (TG, CE) synthesis (DGAT, SOAT, and ATGL) are shown in dark blue (left). D-PDMP (targeting GCS enzymatic activity and HexCer levels in plasma membrane) and avasimibe (targeting SOAT and GCS) negatively affect the initial steps in viral entry (right). (Lower part of the figure) Lipids upregulated upon MV infection are indicated by red arrows; downregulated lipids are indicated by blue arrows. The dark grey rectangle highlights the sphingolipids along the *de novo* synthesis pathway that are crucial for MV replication. Created by BioRender.com.

This study dissects the specific lipid requirements of target CD4^+^ T cells crucial for viral entry, as well as the functional outcomes of virus-induced lipid remodeling at the post-entry stage (Figure 8). Taken together, our findings demonstrate that MV infection profoundly alters cellular lipid composition during replication. Specifically, it triggers the accumulation of Chol, TGs, and sphingolipids, mainly dhCer and Cer in CD4^+^ T cells at 48 and 72 hpi, (Figure 8, bottom). Notably, despite the significant increase in Cer and dhCer levels in infected cells, *in situ* [*d*_*3*_]-palmitate flux lipidomic analysis uncovered a general block in *de novo* sphingolipid synthesis, alongside a shift in [*d*_*3*_]-palmitate incorporation into newly synthesized TG and PC pools. Importantly, the accumulation of TGs, as well as dhCer and Cer (resembling a cellular stress response), supports the production of infectious viral progeny, establishing a pro-viral function for both neutral lipids and sphingolipids. Furthermore, while we observed a downregulation of GCS activity and HexCer levels during viral replication, functional assays established that basal GCS activity, TG production, and presumably also CE levels in the uninfected cells are crucial for MV attachment and glycoprotein-mediated membrane fusion (Figure 8, upper part). This highlights a differential role for HexCer during virus entry and intracellular replication. These data significantly advance our understanding of how MV hijacks and remodels host lipid metabolism to optimize T-cell entry and replication.

In summary, our data demonstrate that MV infection induces a profound reprogramming of CD4^+^ T cell sphingolipid and fatty acid pathways. This shifts lymphocytes toward a ceramide-rich phenotype that mirrors the spleen’s lipid-dependent regulatory architecture. In addition to direct infection, MV-infected cells can activate sphingomyelinases and induce ceramide platforms on uninfected bystander T cells via glycoprotein-mediated contact (Gassert et al., 2009). As shown previously, this process affects immune-synapse formation and blunts T-cell activation (Shishkova et al., 2007; Avota et al., 2010). Here, we demonstrate that this altered fatty-acid and sphingolipid flux supports viral replication in T cells. We hypothesize that once these metabolically rewired CD4^+^ T cells home to the spleen, they amplify the organ’s intrinsic, ceramide-driven immunoregulatory brakes, thereby enabling systemic immunosuppression. Supporting this, histological analysis confirms the infiltration of MV-infected immune cells in lymphoid organs (Allen et al., 2018) and suppressed cell proliferation in the spleen and draining lymph nodes (Niewiesk et al., 1999; Avota et al., 2001). Furthermore, because we detected elevated levels of S1P precursors (dhSph, Sph, and Cer) in MV-infected CD4^+^ T cells, we speculate that infected cells disrupt normal migration by altering S1P gradients. Finally, in line with the concept of lipid-driven inter-organ immunometabolic crosstalk (Tarantino and Citro, 2024), this shared lipid axis suggests that MV-induced metabolic reprogramming can propagate from T cells to the spleen and then outward to peripheral organs such as the skin, liver, gut, and bone marrow.

## Data Availability

The original contributions presented in the study are included in the article/Supplementary Material, further inquiries can be directed to the corresponding author.
