# Supplementary material for: Measles virus reprograms CD4+ T-cell sphingolipid and fatty acid metabolism to facilitate infection
Source: Front Cell Dev Biol. 2026 Aug 19;14:1938931. doi: 10.3389/fcell.2026.1938931 (PMC13534112; doi:10.3389/fcell.2026.1938931)
Supplement: Supplementary file 1 [file Supplementaryfile1.pdf]

Supplementary Material

1 Supplementary Figures and Table

1.1 Supplementary Figures

A

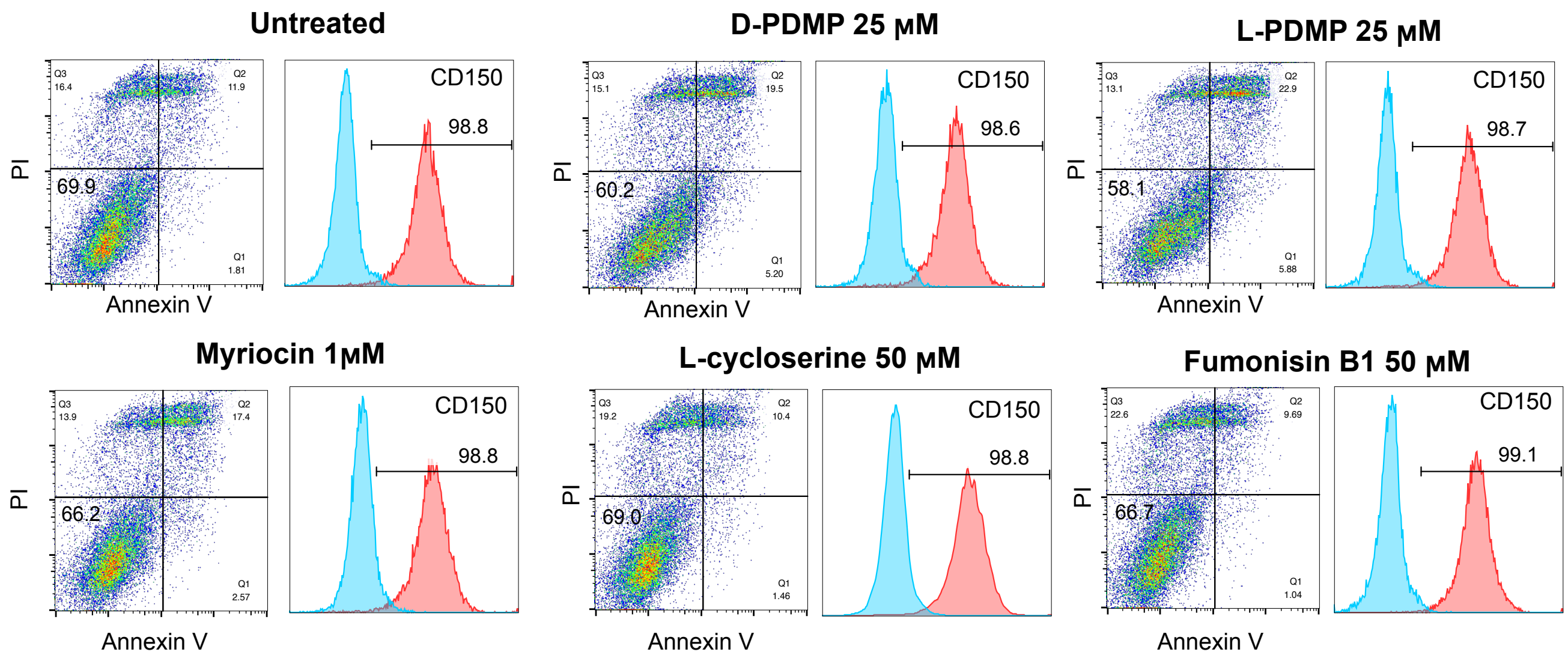
Untreated
D-PDMP 25 μM
L-PDMP 25 μM
Myriocin 1μM
L-cycloserine 50 μM
Fumonisin B1 50 μM
PI
Annexin V
CD150
69.9
98.8
60.2
98.6
58.1
98.7
66.2
98.8
69.0
98.8
66.7
99.1

B

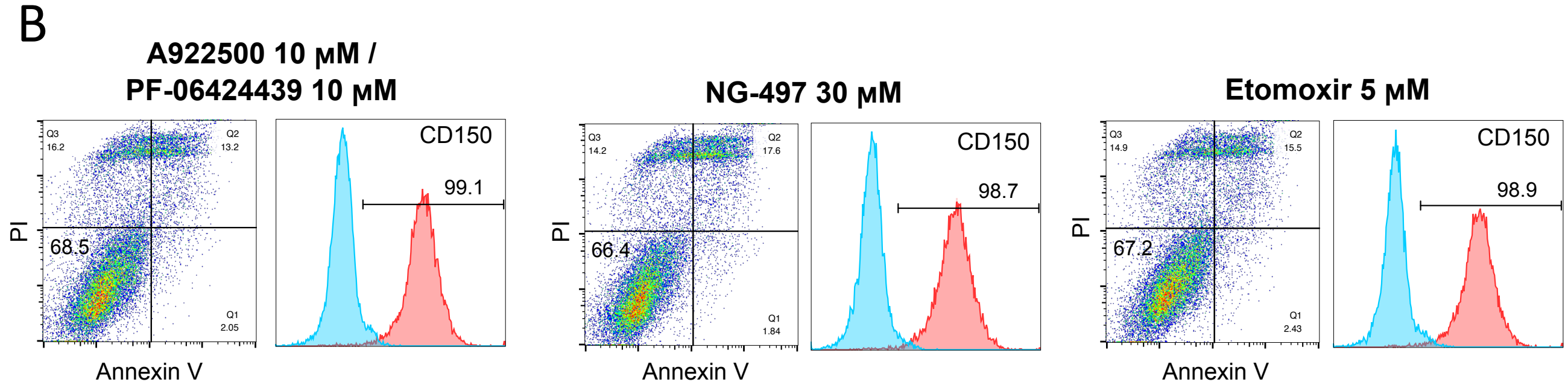
A922500 10 μM /
PF-06424439 10 μM
NG-497 30 μM
Etomoxir 5 μM
PI
Annexin V
CD150
68.5
99.1
66.4
98.7
67.2
98.9

C

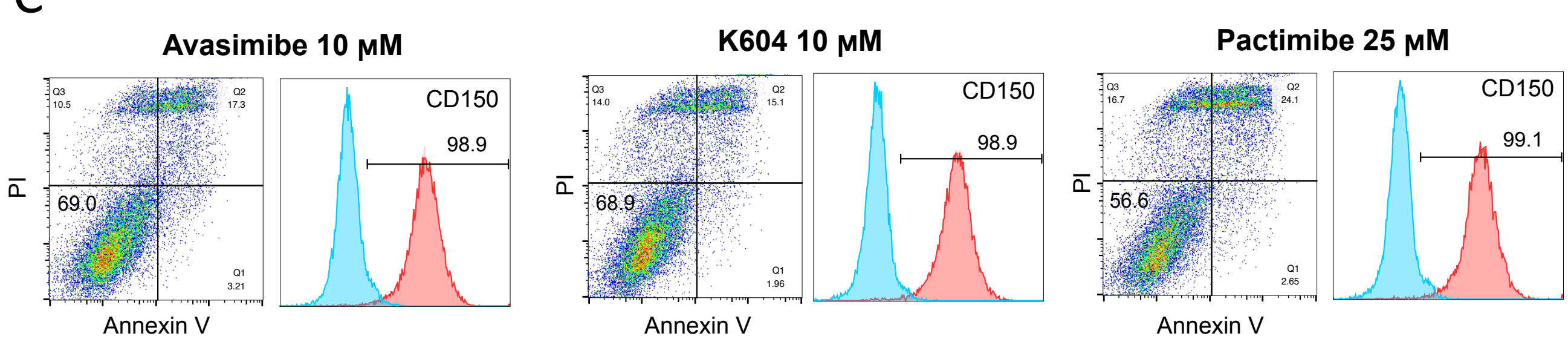
Avasimibe 10 μM
K604 10 μM
Pactimibe 25 μM
PI
Annexin V
CD150
69.0
98.9
68.9
98.9
56.6
99.1

D

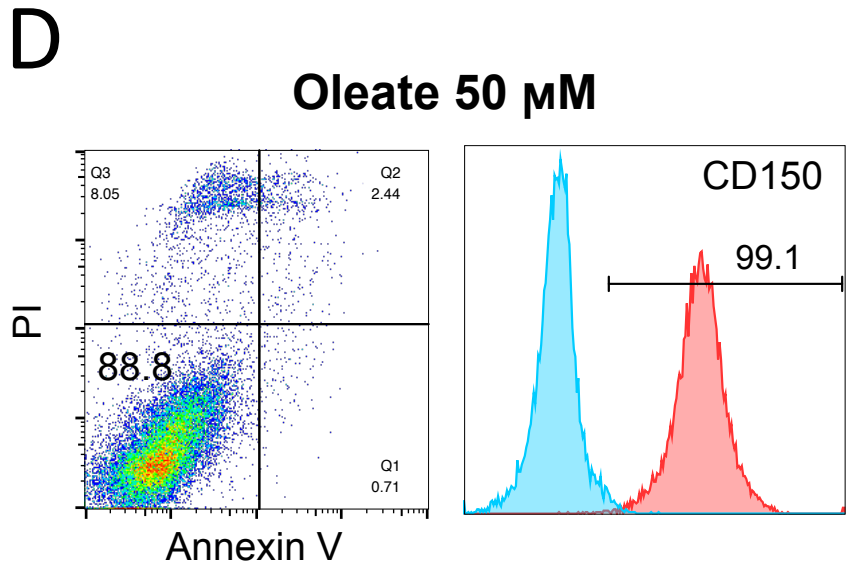
Oleate 50 μM
PI
Annexin V
CD150
88.8
99.1

Supplementary Figure S1. Inhibitor treatments do not alter cell viability or surface expression of the MV entry receptor CD150.

Representative flow cytometry analysis showing cell viability (dot plots) and CD150 surface expression (histograms) in primary $CD4^+$ T cells that were either left untreated or treated with the indicated inhibitors. Cells were treated overnight with inhibitors targeting enzymes involved in (A) *de novo* sphingolipid synthesis, (B) triacylglycerol synthesis and mobilization, (C) cholesteryl ester synthesis pathways or (D) cells were supplemented with oleate. The percentage of viable cells (Annexin V^-/PI^-) are indicated within the lower-left quadrants of the dot plots. Numbers within the histograms indicate the percentage of $CD150^+$ cells.

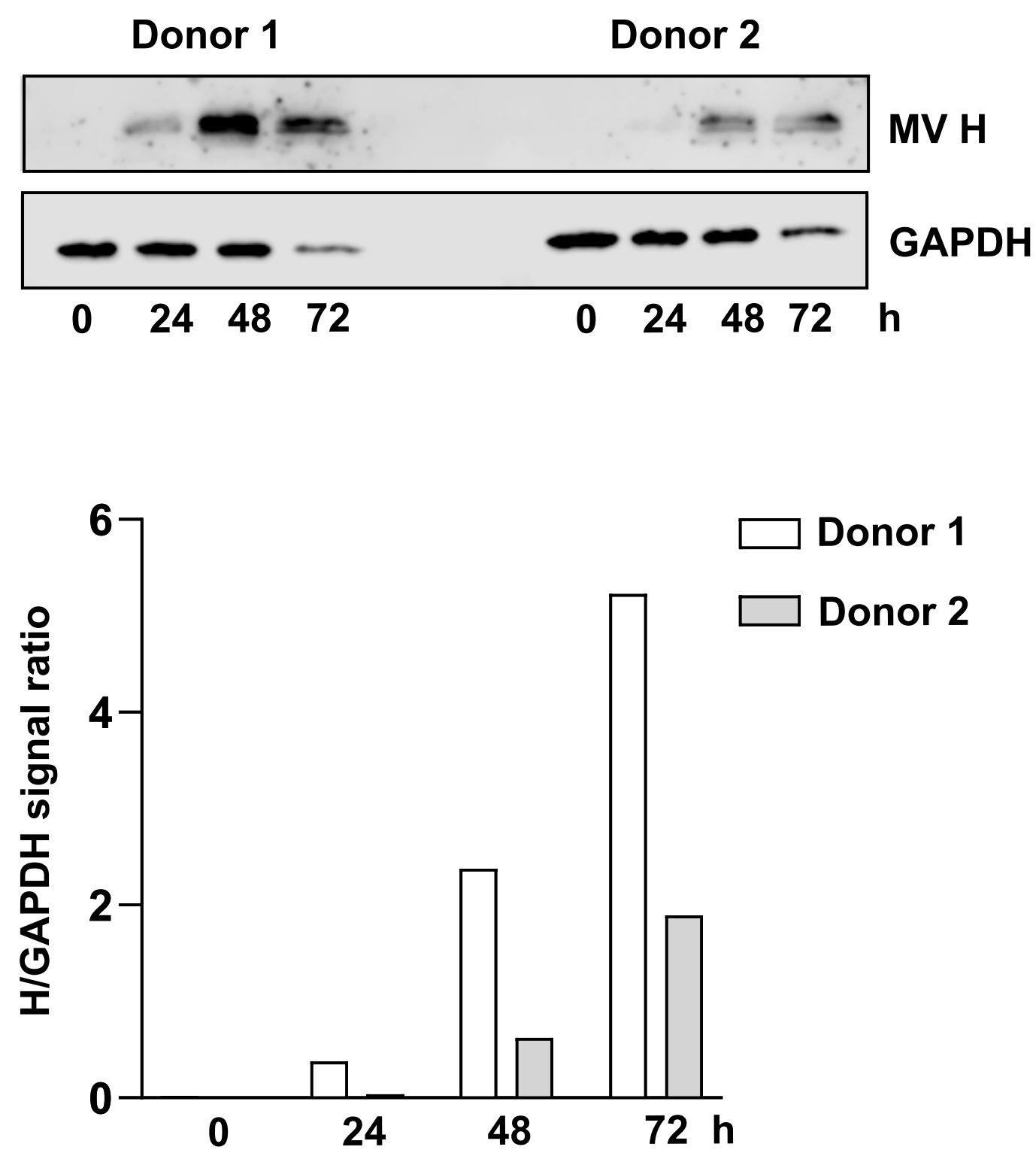

Supplementary Figure S2. Measles virus glycoprotein H expression in infected $CD4^+$ T cells.

(Upper panels) Representative Western blot analysis of MV H protein levels in uninfected (0 h) and MV-infected $CD4^+$ T cells isolated from two independent donors at the indicated time points post-infection. Glyceraldehyde-3-phosphate dehydrogenase (GAPDH) was used as the housekeeping control and internal loading reference. (Lower panel) Densitometric quantification of MV H protein expression normalized to the GAPDH signal.

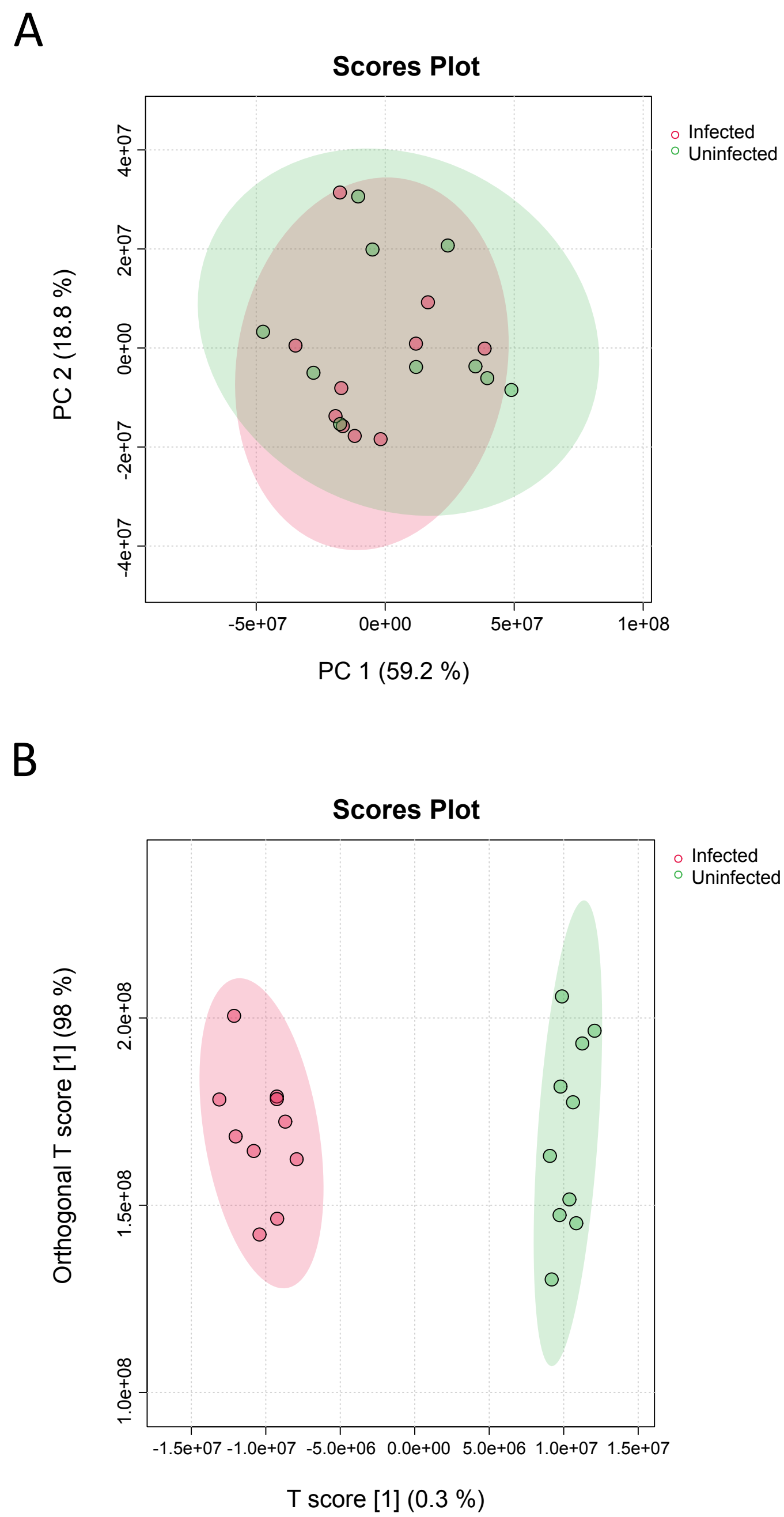

Supplementary Figure S3. Multivariate statistical analysis of the CD4^{+} T cell lipidome following Measles Virus (MV) infection.

(A) Unsupervised principal component analysis (PCA) score plot of untargeted lipidomic data obtained from MV-infected (pink) and uninfected (green) primary human CD4^{+} T cells at 72 hpi. (B) Supervised orthogonal partial least squares discriminant analysis (OPLS-DA) score plot demonstrating clear discrimination between infected and uninfected cohorts. Individual data points represent individual replicates (n = 10).

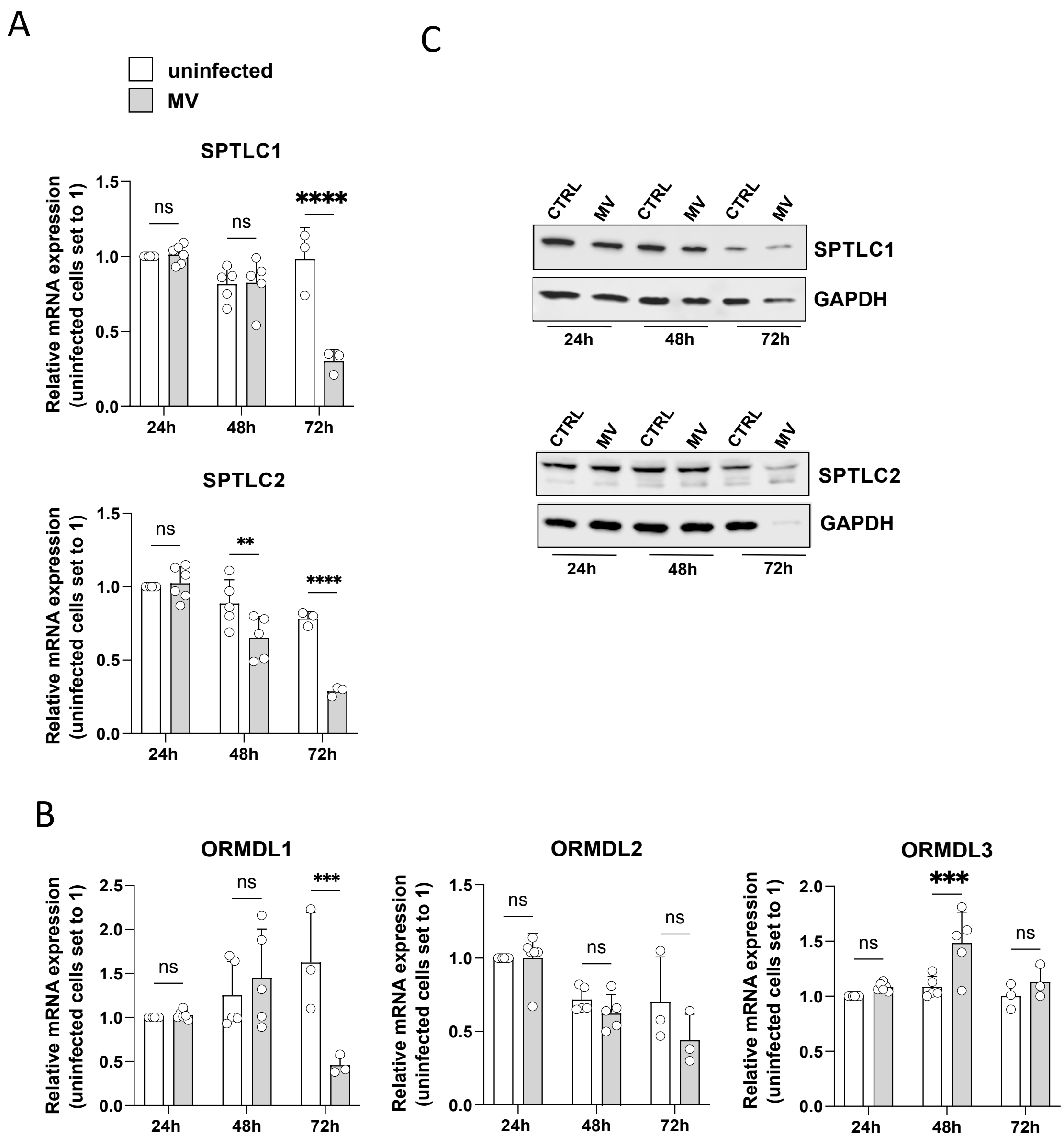

Supplementary Figure S4. Expression of the serine palmitoyltransferase (SPT) complex in MV-infected primary CD4⁺ T cells.

(A-B) Relative mRNA expression of the SPT complex catalytic (A) and regulatory (B) subunits in $CD4^+$ T cells following MV infection (MOI=0,4) at the indicated time points, as determined by RT-qPCR. Data were normalized to *RNASE P* expression and represent the mean ±SD of three or more independent experiments. Individual donor experiments are indicated by circles. Statistical significance was determined using a two-way ANOVA followed by Šidák's multiple comparisons test versus the respective uninfected control (** < 0.01, *** $p < 0.001$, **** $p < 0.0001$). (C) Representative Western blot images showing protein levels of the SPT complex catalytic subunits SPTLC1 and SPTLC2 in uninfected (CTRL) and MV-infected $CD4^+$ T cells at the indicated time points post-infection. Glyceraldehyde-3-phosphate dehydrogenase (GAPDH) was used as the housekeepingcontrol and internal loading reference.

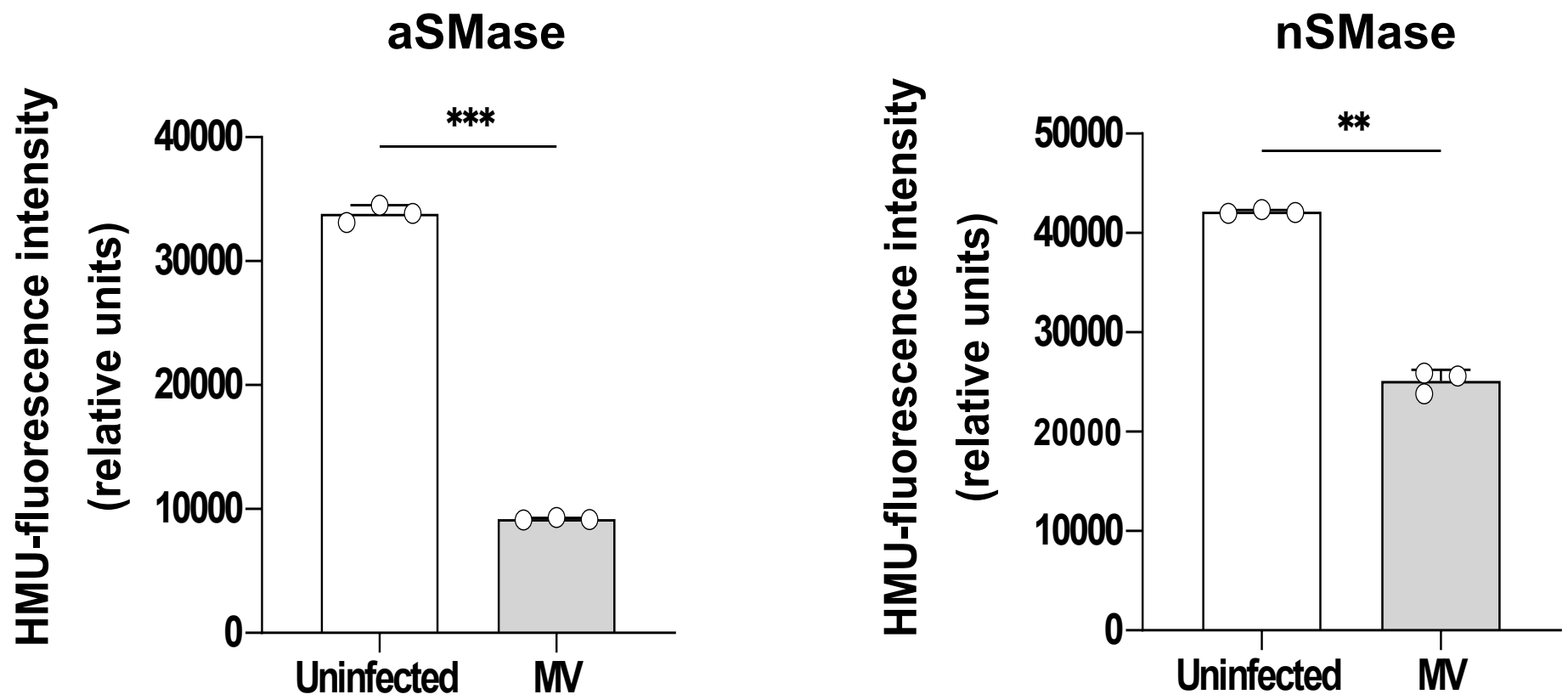

Supplementary Figure S5. Enzymatic activities of acid and neutral sphingomyelinases are reduced following MV infection.

Quantitative analysis of acid sphingomyelinase (aSMase) and neutral sphingomyelinase (nSMase) activities in cell lysates of primary CD4^{+} T cells at 72 hpi. Data are presented as mean ±SD of three independent donor experiments. Circles within the graphs indicate individual donor values. Statistical significance was determined using a paired, two-tailed Student's t-test (** < 0.01, *** p < 0.001).

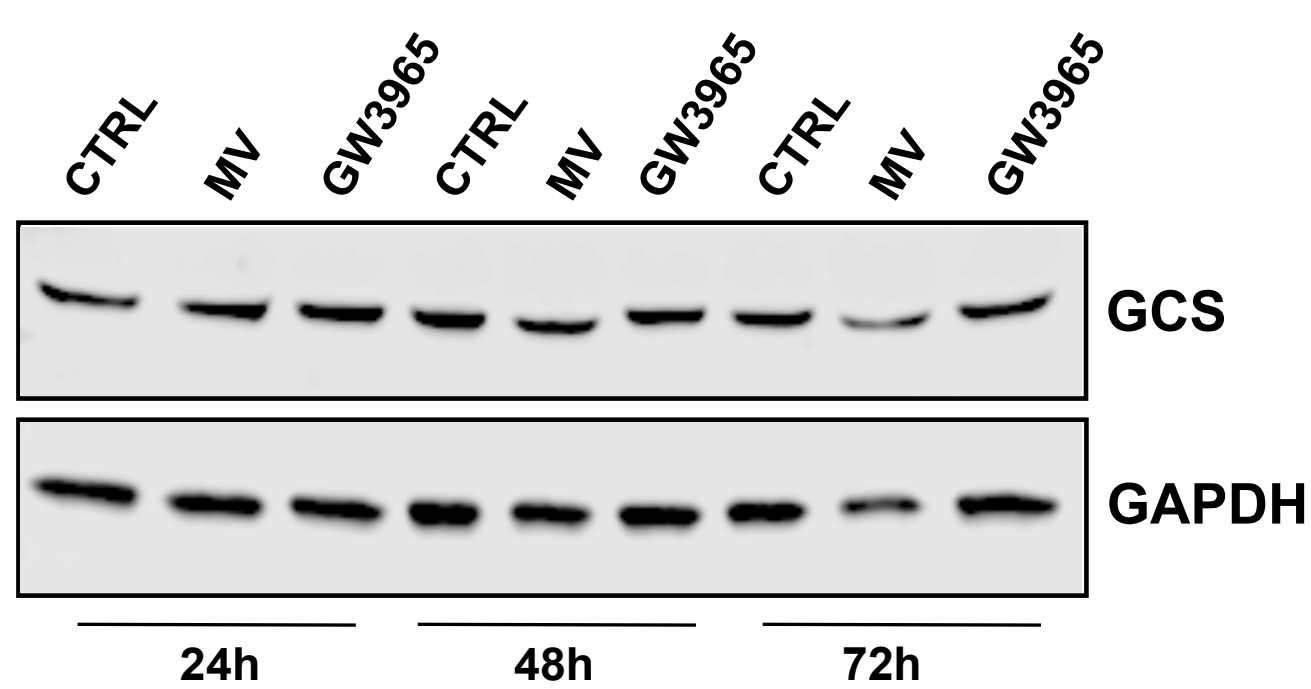

Supplementary Figure S6. Glucosylceramide synthase expression remains unaffected in MV-infected cells.

Representative Western Blot analysis of glucosylceramide synthase (GCS) protein levels in primary CD4^{+} T cells at the indicated time points post-MV infection. Glyceraldehyde-3-phosphate dehydrogenase (GAPDH) served as the internal loading control. Cells treated with 1µM GW3965, a liver X receptor (LXR) agonist known to positively regulate GCS expression, were included as a positive control to confirm GCS induction and validate antibody detection specificity.

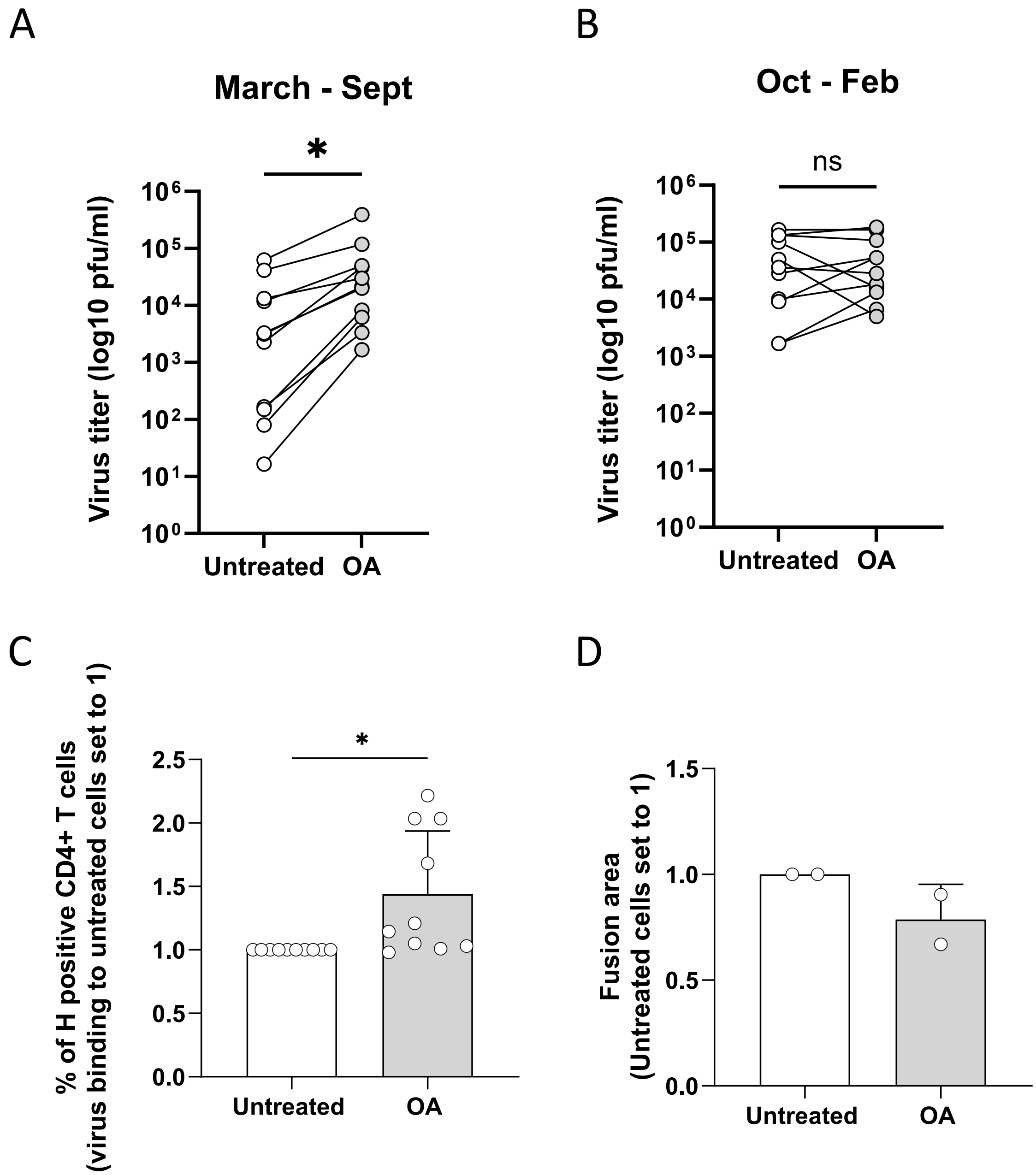

Supplementary Figure S7. Oleate (OA) supplementation promotes measles virus replication.

(A, B) Infectious virus yields in primary $CD4^{+}$ T cells isolated from donors during (A) March to September and (B) October to February. Lines connect the data points of untreated and OA-treated cell cultures originating from the same individual donor. (C) Quantification of viral binding by flow cytometry and (D) relative fusion area determined via microscopic analysis following pretreatment with oleate (OA). Data in C and D represent mean ± SD, with circles indicating independent experiments. Statistical significance was determined using an unpaired, two-tailed Student's t-test (* $p < 0.05$; ns, non-significant).

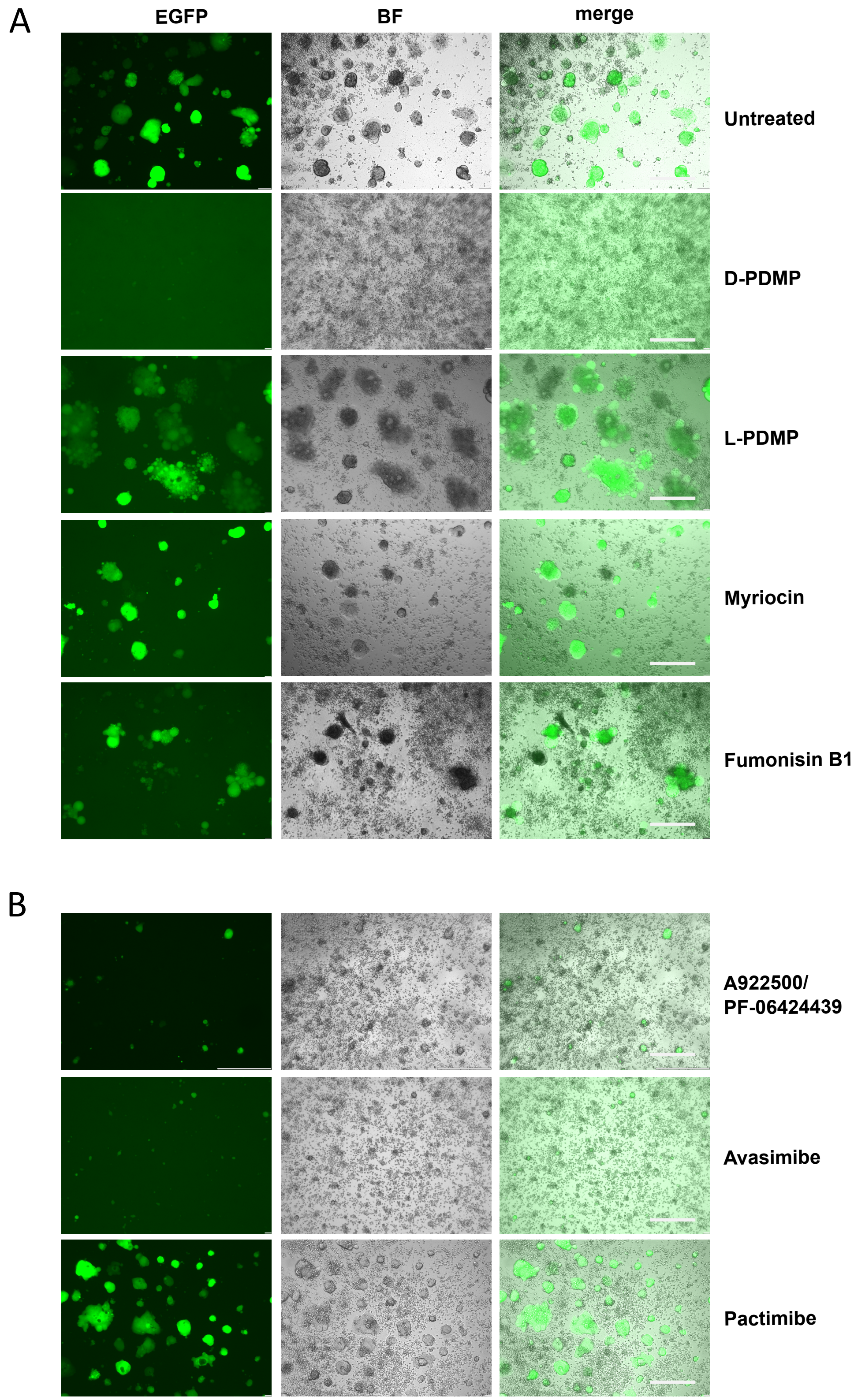

Supplementary Figure S8. Inhibition of glucosylceramide synthase or diacylglycerol O-transferases reduces the number of infected cells.

Representative fluorescence microscopy images of primary $CD4^+$ T cells infected with MV IC323-

EGFP (MOI=0.4) and imaged at 72 hpi. Cells were either left untreated or pre-treated prior to infection with inhibitors targeting enzymes along (A) the *de novo* sphingolipid biosynthesis pathway or (B) the neutral lipid synthesis pathways. The specific molecular targets of these inhibitors are schematically depicted in the upper panels of Figures 6C, 6D and 6E. Scale bar: 300 µm.

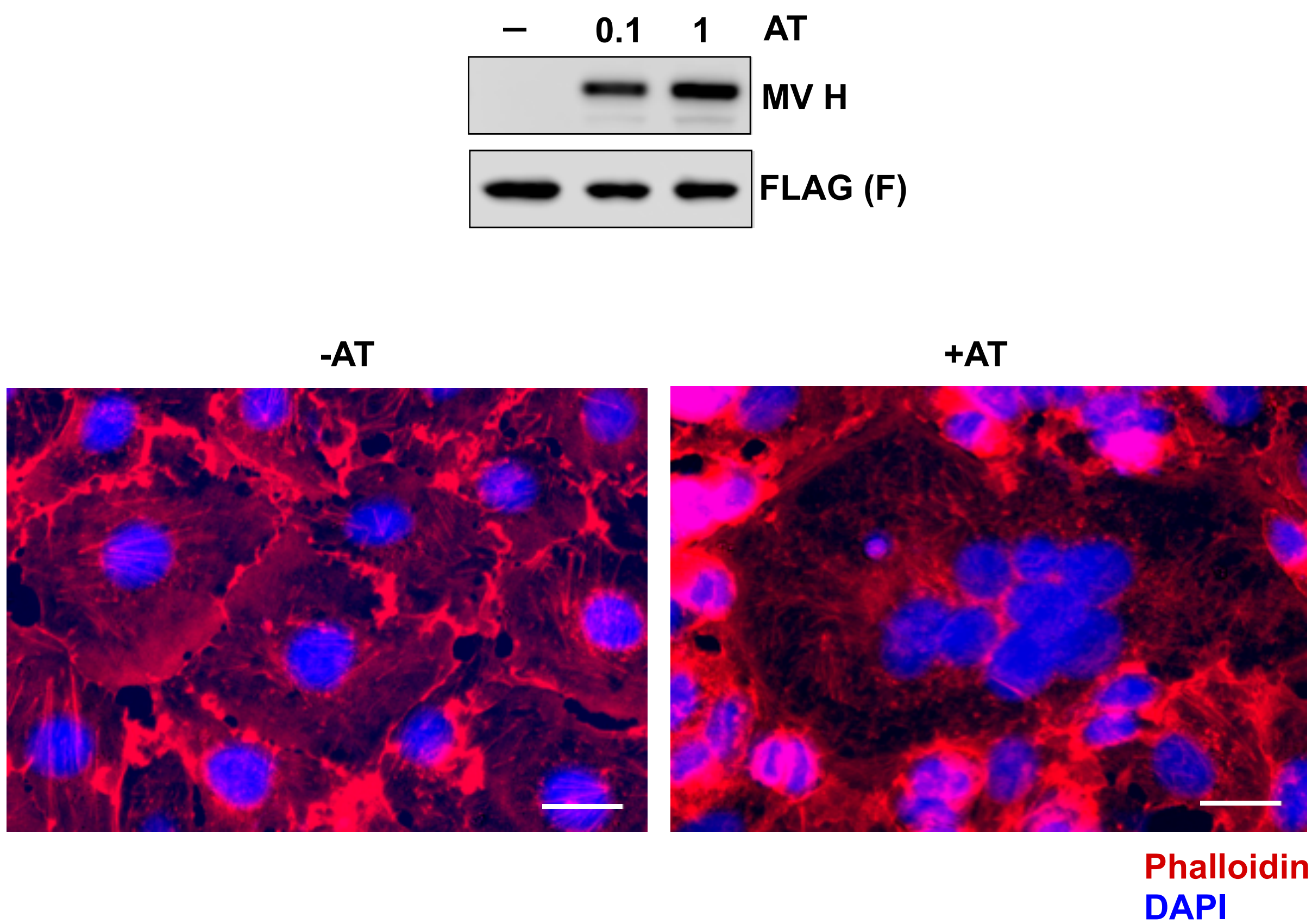

Supplementary Figure S9. Measles virus glycoprotein F- and H-mediated cell-to-cell fusion assay.

(Upper panels) Western blot analysis of HEK293 cells stably expressing MV F-FLAG and tetracycline-inducible H protein (indH). Cells were either left untreated or treated with the indicated concentrations (µg/mL) of anhydrotetracycline (AT) for 24 hours prior to cell lysis, followed by immunoblotting with anti-H and anti-FLAG antibodies. (Lower panels) Representative immunofluorescence microscopy images of the fixed and permeabilized co-cultures containing CD150-expressing cells (VERO-SLAM) and MV H/F expressing cells (HEK F/indH). Cocultures were either left untreated (-AT) or treated with AT for 6 hours (+AT). The F-actin of cytoskeleton was counterstained with fluorescent Phalloidin, and nuclei were visualized with DAPI. Cell-to-cell fusion is indicated by the formation of multinucleated giant cell in the +AT condition. Scale bar: 5 µm.

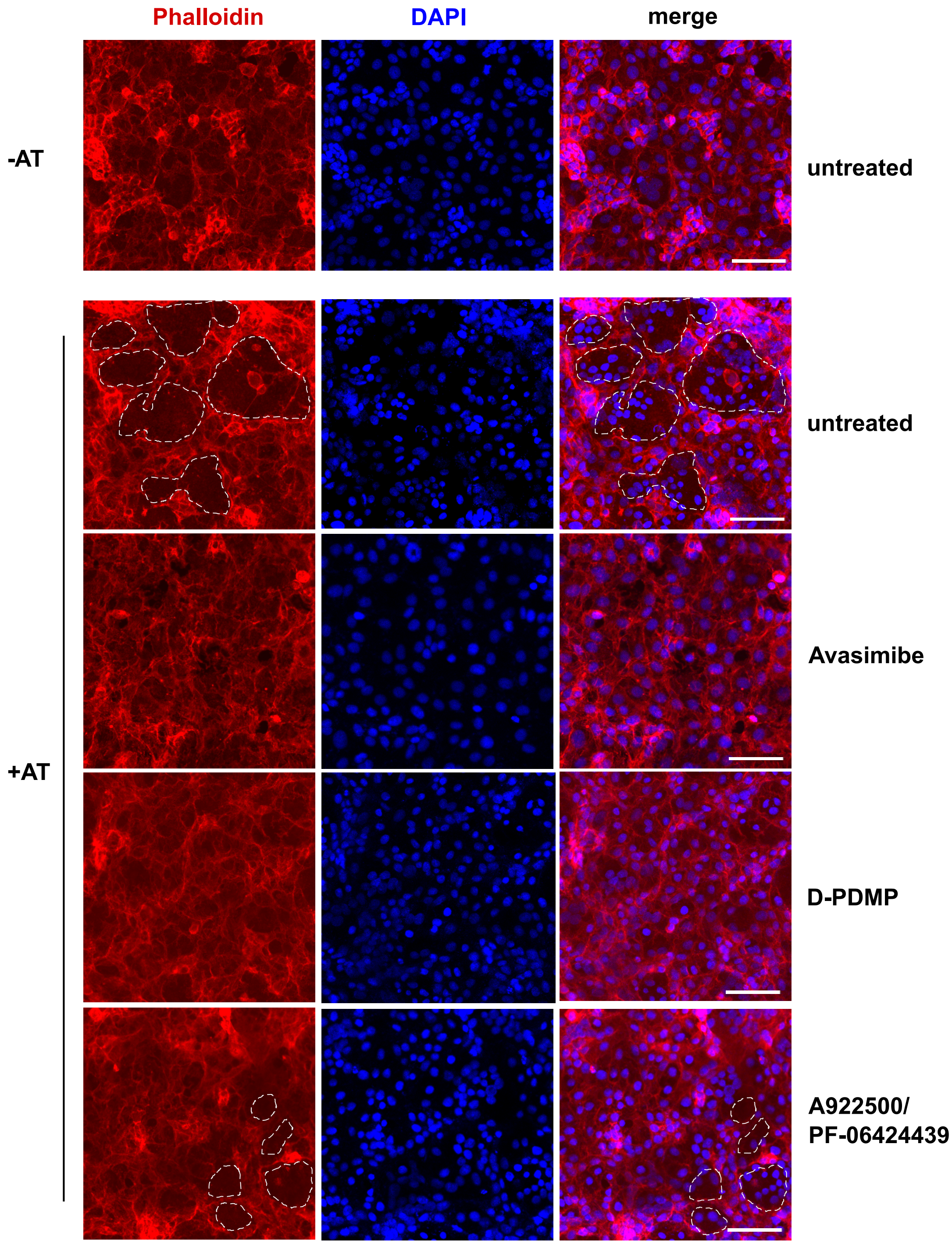

Supplementary Figure S10. Inhibition of glucosylceramide synthase or diacylglycerol O-transferases impairs cell-to-cell fusion.

Representative immunofluorescence microscopy images of VERO-SLAM and HEK F/indH cell co-cultures. Cells were either left untreated (upper panels) or treated with anhydrotetracycline (AT) alone or in combination with the indicated inhibitors for 6 hours (+AT). Co-cultures were stained for F-actin (red) and nuclei (blue). Multinucleated regions are outlined by dashed lines. Scale bar: 100 µm.

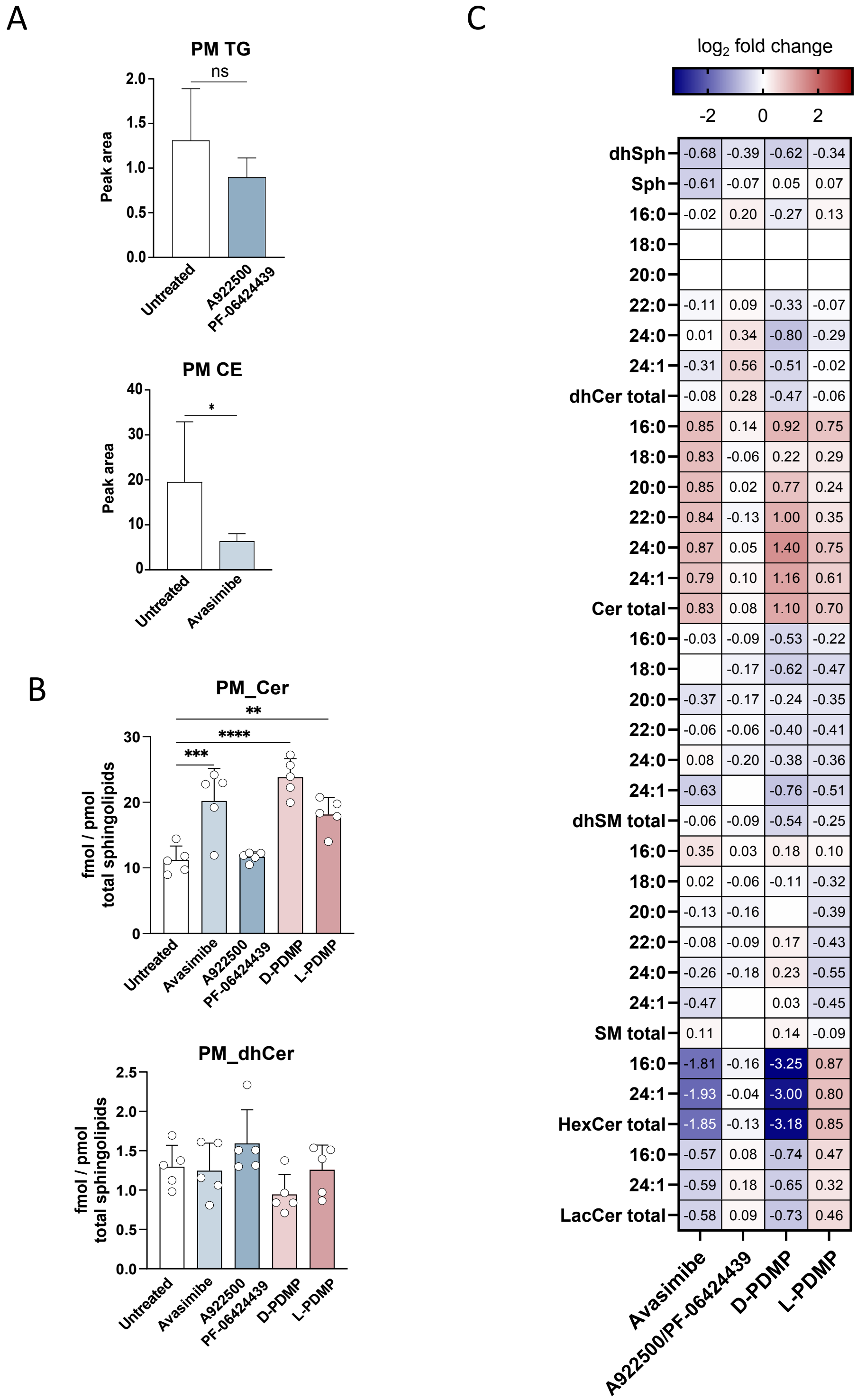

Supplementary Figure S11. The SOAT inhibitor avasimibe and the glucosylceramide synthase inhibitor D-PDMP alter plasma membrane sphingolipid content in a similar manner.

Primary CD4^{+} T cells were either left untreated or treated for 24 hours with inhibitors targeting triacylglycerol (TG) synthesis (A922500, PF-06424439), cholesteryl ester (CE) synthesis (avasimibe), or HexCer levels (D-PDMP, L-PDMP) prior to plasma membrane (PM) isolation and lipid extraction.

(A) The levels of TG (upper graph) and CE (lower graph) measured in the PM fraction by UPLC-qTOF-MS (n=4). Data are presented as mean ± SD (relative to untreated control). (B-C) Sphingolipids (SLs) quantified in samples via LC-MS/MS (n=5). Panel (B) shows changes in PM Cer and dhCer levels in inhibitor-treated cells relative to the total SL pool (fmol/pmol total SLs). Data represent mean ± SD relative to the untreated control, with circles indicating individual donor samples. (D) Heatmap displaying $\log_2$ fold-change values of specific SL species in inhibitor-treated cells relative to the untreated control. Statistical significance compared to the untreated control (white bar) was determined using an ordinary one-way ANOVA followed by Dunnett's post-hoc test (* < 0.05, ** < 0.01, *** p < 0.001, **** p < 0.0001). dhSph, dihydrosphingosine; Sph, sphingosine; Cer, ceramide; dhCer, dihydroceramide; dhSM, dihydrosphingomyelin; SM, sphingomyelin; HexCer, Hexosylceramide; LacCer, lactosylceramide.

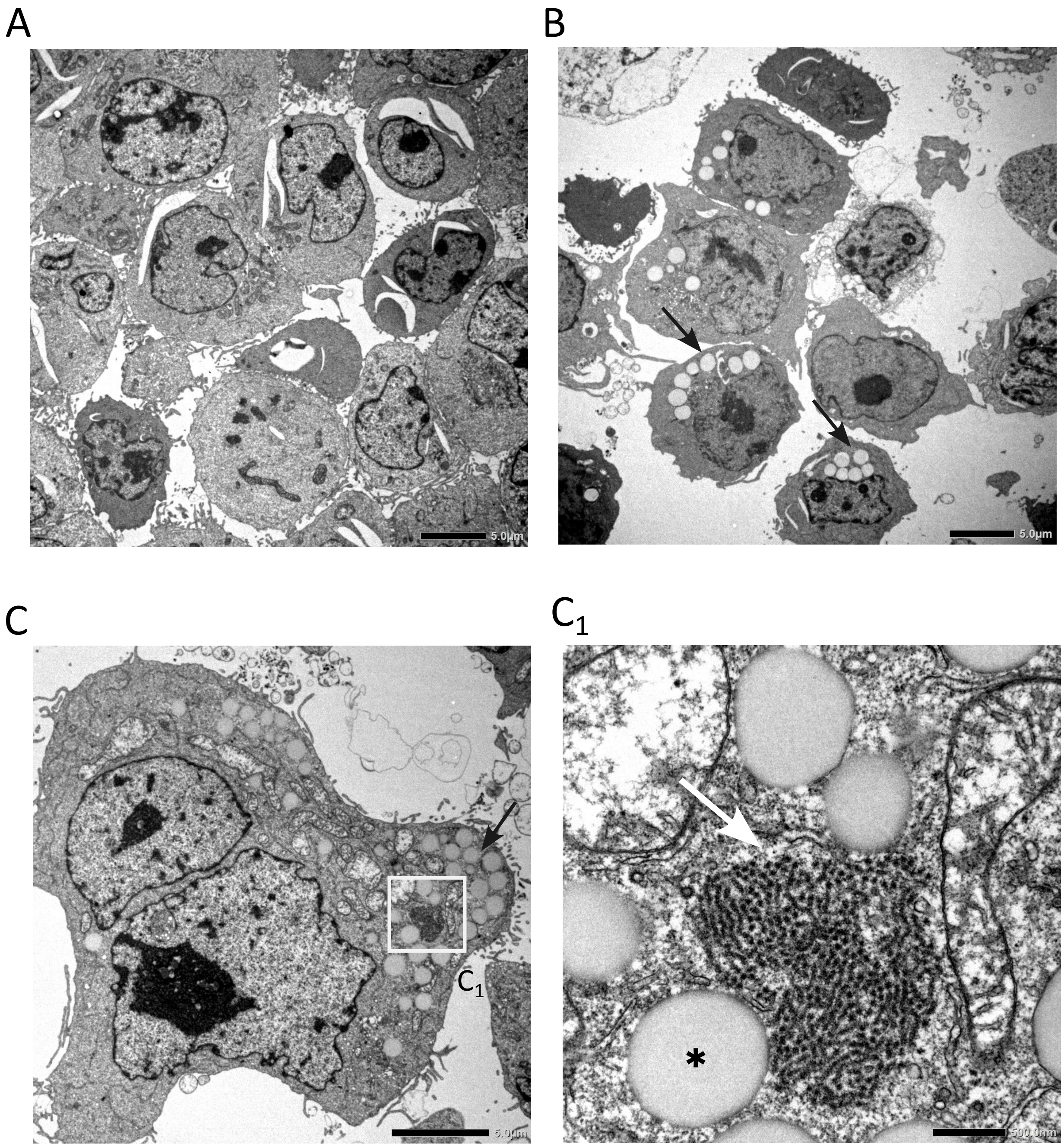

Supplementary Figure S12. MV infection induces lipid droplet formation in primary $CD4^+$ T cells.

(A-C_1) Representative transmission electron microscopy (TEM) images of (A) uninfected and (B-C_1) MV infected primary $CD4^+$ T cells at 24 hpi. (B) Black arrows indicate lipid droplets. (C) Syncytium formation demonstrating the fusion of two infected cells (two nuclei visible). The white rectangular demarcates the region magnified in panel C_1. (C_1) Enlarged inset of panel C highlighting lipid droplet (asterisk) in close proximity to a cytoplasmic MV inclusion body containing distinct tubular nucleocapsid structures (white arrow).

1.2 Supplementary Table

	Ionform	Compound	m/z	Retention time [min]	Anova (p)	Fold Change	Minimum CV [%]
**TG 54:5**	[M+NH4]+	9.87_898.7874m/z+	898.787	9.87	2.71E-08	4.60	15
**Cer 34:1;O2**	[M+OAc]-	7.77_537.5118n-	596.526	7.77	1.27E-08	3.06	18
**TG 56:7**	[M+NH4]+	9.80_921.7814n+	922.789	9.80	2.47E-08	8.21	15
**TG 54:6**	[M+NH4]+	9.70_896.7725m/z+	896.773	9.70	5.49E-08	7.32	21
**PC34:1**	[M+H]+	7.75_759.5778n+	760.585	7.75	5.76E-06	0.68	12
**PC36:1**	[M+H]+	8.13_787.6095n+	788.617	8.13	7.53E-05	0.62	16
**PC36:2**	[M+H]+	7.79_768.5675n+	786.601	7.79	7.65E-08	0.63	10
**PC 32:1**	[M+H]+	7.34_732.5544m/z+	732.554	7.34	7.96E-08	0.56	11
**PC 30:0**	[M+H]+	7.27_706.5396m/z+	706.540	7.27	1.01E-06	0.47	12

Supplementary Table 1. Analytical and statistical data of key lipid features separating MV-infected and uninfected cells obtained by UPLC-qTOF-MS.
